# Perceptions of healthcare quality in Ghana: Does health insurance status matter?

**DOI:** 10.1371/journal.pone.0190911

**Published:** 2018-01-16

**Authors:** Stephen Kwasi Opoku Duku, Edward Nketiah-Amponsah, Wendy Janssens, Menno Pradhan

**Affiliations:** 1 Noguchi Memorial Institute for Medical Research, University of Ghana, Accra, Ghana; 2 Faculty of Economics and Business Administration, Free University of Amsterdam, Amsterdam, The Netherlands; 3 Amsterdam Institute for Global Health and Development, Amsterdam, The Netherlands; 4 Economics Department, University of Ghana, Legon- Accra, Ghana; Public Library of Science, UNITED KINGDOM

## Abstract

This study’s objective is to provide an alternative explanation for the low enrolment in health insurance in Ghana by analysing differences in perceptions between the insured and uninsured of the non-technical quality of healthcare. It further explores the association between insurance status and perception of healthcare quality to ascertain whether insurance status matters in the perception of healthcare quality. Data from a survey of 1,903 households living in the catchment area of 64 health centres were used for the analysis. Two sample independent t-tests were employed to compare the average perceptions of the insured and uninsured on seven indicators of non-technical quality of healthcare. A generalised ordered logit regression, controlling for socio-economic characteristics and clustering at the health facility level, tested the association between insurance status and perceived quality of healthcare. The perceptions of the insured were found to be significantly more negative than the uninsured and those of the previously insured were significantly more negative than the never insured. Being insured was associated with a significantly lower perception of healthcare quality. Thus, once people are insured, they tend to perceive the quality of healthcare they receive as poor compared to those without insurance. This study demonstrated that health insurance status matters in the perceptions of healthcare quality. The findings also imply that perceptions of healthcare quality may be shaped by individual experiences at the health facilities, where the insured and uninsured may be treated differently. Health insurance then becomes less attractive due to the poor perception of the healthcare quality provided to individuals with insurance, resulting in low demand for health insurance in Ghana. Policy makers in Ghana should consider redesigning, reorganizing, and reengineering the National Healthcare Insurance Scheme to ensure the provision of better quality healthcare for both the insured and uninsured.

## Introduction

### Background

Healthcare financing in Sub-Saharan Africa (SSA) is a grim scenario. SSA comprises 12% of the world’s population and 22% of the total global disease burden [1], yet accounts for only 1% of the world’s health expenditure and 2% of the global workforce in healthcare. With low per capita income, limited domestic revenue mobilisation, and ineffective health systems, countries in SSA are ill-prepared to effectively address health financing problems [2]. Governments in most SSA countries are not the sole financiers of healthcare. In these cases, more than half of the health expenditure is financed through out-of-pocket payments, which places financial burdens on households and serves as a barrier to healthcare access [3, 4].

Countries in SSA, such as South Africa, Gabon, Mali, Senegal, Uganda, Tanzania, Nigeria, and Ghana have taken steps towards universal coverage by adopting risk pooling systems to provide financial protection, particularly to the poor and vulnerable in their societies [5–9]. These risk-pooling systems are either small, community-based health insurance (CBHI) schemes or social health insurance (SHI) schemes. The CBHI schemes are often voluntary and limited in geographic scope to a few rural communities with small numbers of enrolees as well as limited benefit coverage. The SHI schemes, on the other hand, have a wide scope, covering all regions in a country with subsidised premiums, government funding, and exemption policies. In some SHI schemes, enrolment is mandatory by law but voluntary in practice, as in Ghana. The benefit package covers quite comprehensive outpatient and inpatient healthcare services and medications [10, 11]. Surprisingly, enrolment in most of these SHI schemes remains low even though premiums are highly subsidised. In Ghana, as of December 2013, active membership in its National Health Insurance Scheme (NHIS) was 38% of the population [12]. People’s perception about the NHIS and healthcare quality has been identified as one of the factors that informs their decision to enrol or drop out of the scheme [13,14].

Prior economic literature has stipulated that the demand for health insurance is dependent on the quality of healthcare and assumes that quality is a constant, independent of health insurance status [15, 16]. The evidence on the relationship between health insurance enrolment and perceived quality of healthcare is very limited. Jehu-Appiah et al [14] found that the perceptions related to providers, schemes and community attributes play an important role at varying extents in household decision to voluntarily enrol and remain enrolled in insurance schemes. A systematic review by Spaan et al. [17] of the impact of health insurance in Africa and Asia, concluded that there is a weakly positive effect of SHI and CBHI on quality of healthcare and that the effect of health insurance on quality of healthcare is woefully under researched. However, recent qualitative evidence suggests that perceived quality of healthcare is not the same across insured and uninsured patients [18, 19]. A recent study by Robyn et al. [20], has also indicated that perceived quality of healthcare may be dependent on health insurance status, although they used a small sample of 398 patients to assess the effect of insurance status on technical quality and overall patient satisfaction.

This study builds on the work of Robyn et al. [20] to offer an explanation for the low demand for health insurance enrolment in Ghana, examining a larger sample and focusing on non-technical quality of care indicators (‘soft’ dimensions of quality), such as information provision, complaint lodging, and waiting times, instead of medical technical quality. The focus on non-technical quality of care stems from three main reasons. First, the NHIS accreditation process pays attention to medical technical quality and not non-technical quality. Second, the normative perspective of quality suggests that clients’ perceptions of quality are inherently meaningful and should be the primary focus of attention for quality assessments within healthcare systems. Finally, clients’ perceptions affect outcomes such as a health plan or health provider choice, adherence to medical advice, complaints, and grievances; as well as health outcomes, which are powerful drivers important to stakeholders in the health sector [21–23].

We argue that enrolment levels in Ghana are low because there is a perception that service quality is lower for people with health insurance compared to those who make out-of-pocket payments. This study assesses whether the perceptions on healthcare quality depend on health insurance status by analysing cross-sectional household survey data of a random sample representative of 16 districts in two regions in Ghana. The survey included 1,903 household heads. Two-sample independent t-tests were used to compare the average perceptions of quality per health facility between insured and uninsured respondents. A generalised ordered logit regression, controlling for socio-economic characteristics and clustering at health facility level, was done to test the association between insurance status and perceived quality of healthcare.

### The health system and NHIS financing in Ghana

The responsibility of the Ministry of Health in Ghana is to provide policy guidance, regulation, and strategic direction to service providers, regulatory bodies, and the National Health Insurance Authority (NHIA) [24, 25]. The service providers include Ghana Health Service (responsible for service delivery in all public health facilities), non-governmental organizations (e.g., Christian Health Association of Ghana), and private providers. There are however more private health facilities in the urban areas as compared to the rural areas and vice versa. The quality of healthcare in private health facilities is generally purported to be higher than public ones, whiles the public facilities are often regarded as providing equitable and evidence based healthcare. For this reason, the rich who can afford out-of-pocket payments and private health insurance often patronize private facilities while the poor, mostly in rural areas patronize public facilities [26]. The NHIA and other regulatory bodies (e.g., the. Medical & Dental Council) assess and regulate standards of service quality. The NHIA accreditation process assesses the technical quality of the health facilities before their accreditation to provide healthcare services. These technical assessments focus on medical technical quality with no attention to non-technical quality indicators such as interpersonal relations, queuing systems, complaints, and waiting times [25].

The NHIS was established in 2004 to ensure financial access to equitable and acceptable quality of essential healthcare benefits for all residents in Ghana. The scheme is financed mainly through the National Health Insurance Fund (NHIF). The NHIF monies come from 2.5% of the 17.5% value-added tax (VAT), 2.5% of the 17.5% Social Security and National Insurance Trust (SSNIT), contributions from formal sector employees, accruals to the fund from investments of the NHIA Council, and member contributions from premium payments. The annual premium is set at the NHIS district office level and is dependent on the poverty and economic activity levels of the districts with approval from the NHIS council. The average annual premium is about GH₵25 ($13.14 at an exchange rate of GH₵1 = $1.87 as at December, 2012). The Government of Ghana also allocates funds to the NHIF through parliament and other donor funds [27–30, 24]. The NHIS has a broad benefit package, covering 95% of the burden of diseases in Ghana. The NHIS covers out-patient services (consultation including reviews for general and specialist outpatient consultations); in-patient services (general and specialist in-patient care); maternity care (including antenatal care, normal or assisted deliveries); eye care (refraction, visual fields, A-scan, keratometry) and oral health (pain relief, drainage, tooth extraction, dental restoration, fillings and temporary dressing). It provides premium exemptions for the elderly (70 years and above), SSNIT pensioners, children below 18 years, indigents, pregnant women, and Livelihood Empowerment Against Poverty (LEAP) beneficiaries [11]. Nevertheless, the NHIS claims account for only 16% of total healthcare expenditure and 30% of public health expenditure, while out-of-pocket spending accounts for 37% of total health spending. This exceeds the World Health Organisation (WHO) suggested threshold of 15–20% for out-of-pocket expenditure for adequate financial protection [25].

At the inception of the NHIS, the payment of healthcare providers was by itemized fee-for-service. However, in 2008, the NHIA reformed the provider payment mechanism and introduced the Ghana Diagnostic Related Groupings (G-DRGs) for services and fee-for-service for medicines at all levels of service delivery. The NHIS financing arrangement is such that NHIS district offices sign purchasing contracts with accredited healthcare providers. These contracts stipulate the prescription of generic drugs for the treatment of specific medical conditions and healthcare providers are to submit claims for services rendered to insured clients to the NHIS district offices for reimbursement.

### Conceptual framework

People’s perception of healthcare quality is the outcome of an evaluation process where expectations are compared to actual experiences and realities of care received [31, 32]. Individuals enrol in health insurance because they expect financial protection from excessive out-of-pocket payments for the provision of quality healthcare. The consensus among researchers is that positive perceptions of healthcare quality lead to increased client satisfaction, and the acknowledgment of value and trust in the healthcare provider [32, 16, 15, 33, 34]. This ultimately affects individuals’ demand for healthcare and hence, health insurance.

**Fig 1:**
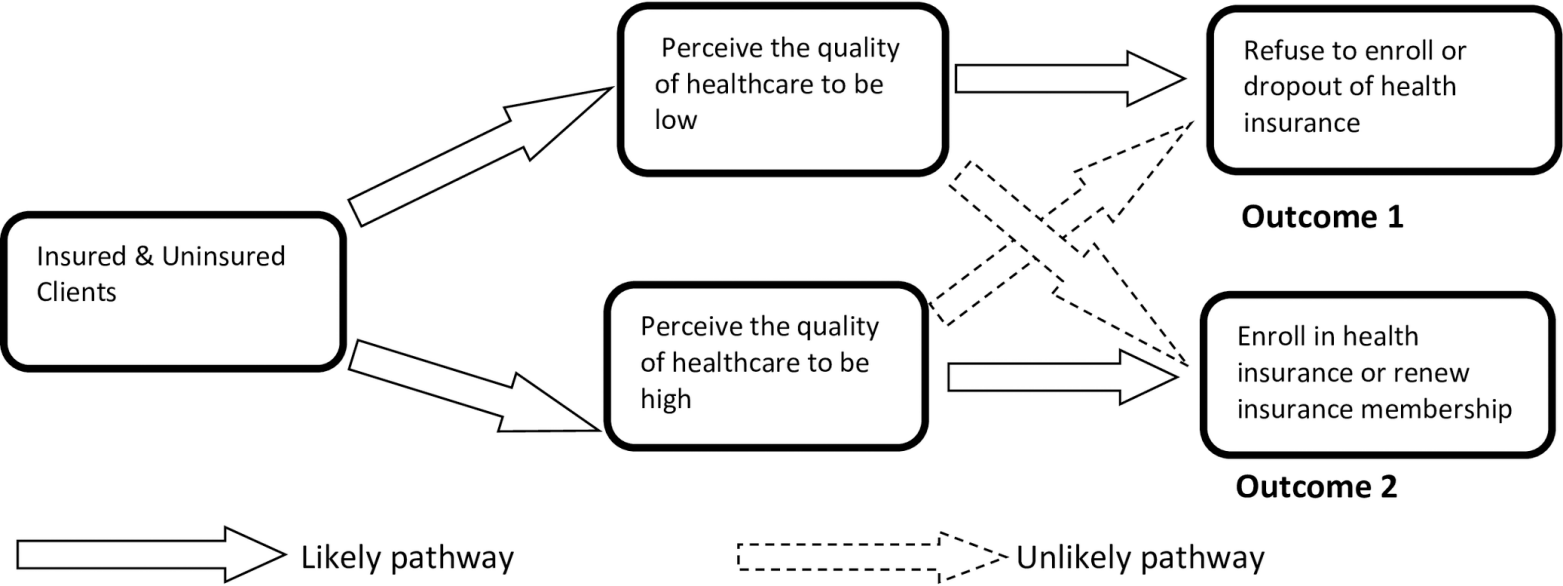
Conceptual framework of independence of quality perception on insurance status.

**Fig 2:**
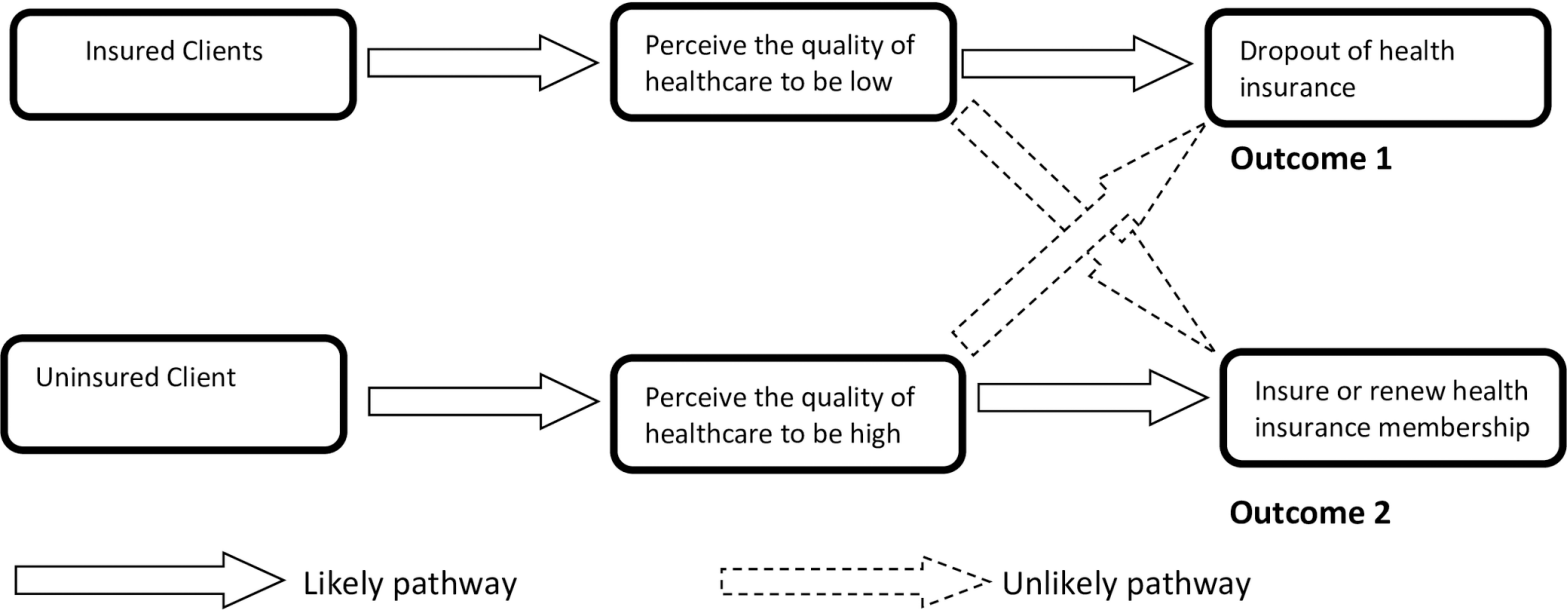
Conceptual framework of dependence of quality perceptions on insurance status.

The utilisation of healthcare is found to be sensitive to quality of care such that households limit their demand when services are of poor quality [35–37] and others bypass low quality health facilities in search of high quality ones [38–41]. The generally accepted interpretation that perception of high quality healthcare influences healthcare utilisation and insurance enrolment assumes that perceptions are independent of insurance status as shown in the “likely pathways” in Fig 1 and ignores a potential dependent effect in the other direction; that is, once insured, people may perceive their healthcare quality as poor depending on the service delivery processes they go through. This may be due to dissatisfaction with the service delivery processes as well as poor technical quality of care provided to the insured. The insured are reported to indicate lower perceived quality of care than the uninsured in recent studies [18, 19]. This paper argues that perception on healthcare quality may be dependent on health insurance status as shown in the likely pathways in Fig 2 below. Hence, ‘enrolment status (insured or uninsured) can influence the direction (low or high) of quality perception’.

If the insured perceive the quality of the healthcare they receive to be low, how does that influence their health insurance renewal decisions. This study contributes the sparse evidence in the literature on the dependence of perceived quality of healthcare on health insurance status.

## Methods

### Study setting

This study is part of a larger research project: the Client-Oriented Health Insurance System in Ghana (COHEiSION) Project. The aim of the research is to identify the main perceived barriers to (re)-enrol in the NHIS, and to design, implement, and evaluate the most effective intervention that can address them. The research was conducted in the Greater Accra and Western regions of Ghana. Ideally, a mix of one northern and one southern region would have provided a much better balance in terms of health insurance enrolment coverage and socio-economic setting. However due to resource and time constraints these two southern regions were selected. These regions are situated in the Eastern and Western coast of Ghana, respectively, with a contrasting difference in urban and rural populations. The Greater Accra Region with Accra, the capital city of Ghana, has a largely urban population of about four million, accounting for 16.3% of the national population. The Western region has a predominantly rural population of about two million, representing 9.6% of the national population [42]. These two regions were purposively selected to provide the rural/urban balance which was of interest to the Project.

### Sampling procedure and data collection

This study analyses data that are representative of 64 communities in 16 districts from the baseline survey of the research project based on a three-stage stratified sampling procedure. At the first stage, 16 districts were purposively selected from the two regions, eight in each region. The second stage involved the purposive selection of 64 primary healthcare facilities (government health centres and private clinics), four in each of the 16 districts. At the third stage, enumeration area (EA) maps were used to list all residential buildings within a 10 km radius of the catchment area of each selected health facility. Simple random sampling was then used to sample 30 households from the catchment area of each of the 64 health facilities to obtain a total sample of 1,920 households.

A baseline household survey was conducted from March to April 2012. The survey used a paper based structured questionnaire, which was translated into the two local languages (Ga and Fante) of the project regions. The questionnaire was administered to household heads by experienced and trained interviewers. The survey collected information on socio-demographics, employment status, consumption expenditure patterns, health status, healthcare utilisation behaviour, dwelling characteristics, NHIS enrolment status, and perceptions of non-technical quality of healthcare.

### Measurement of variables

The dependent variables of interest were perceptions on healthcare quality. These were measured by asking household heads to rate on a five-point Likert scale (1 = very dissatisfied; 2 = dissatisfied; 3 = neutral; 4 = satisfied; 5 = very satisfied or 1 = strongly disagree; 2 = disagree; 3 = neutral; 4 = agree; 5 = strongly agree) their satisfaction or agreement with seven perception questions about the non-technical quality of NHIS accredited primary healthcare facilities. For each respondent, an overall healthcare quality index was calculated as an average score of the seven perceptions of quality indicators. The distribution of the average scores was fit into five quintiles to create a five-level ordinal scale for the overall healthcare quality index. The seven quality indicators were (1) satisfaction with service provision; (2) satisfaction with process of lodging a complaint; (3) satisfaction with information provision; (4) satisfaction with waiting time; (5) agreement with the statement that all prescribed drugs were received from the facility; (6) agreement with the statement that health personnel treated patients with insurance cards equally to those without cards and; (7) agreement with the statement that there was a fair queuing system at the health facility.

The key independent variable of interest was insurance status. This variable was measured by asking respondents to indicate Yes = 1, if they were currently insured in any health insurance scheme, and No = 2, if not. The currently insured are respondents who had valid health insurance cards at the time of the survey and the currently uninsured are those who did not. The currently uninsured were further required to indicate Yes = 1, if they were previously insured in any health insurance scheme and No = 2, if they have never been insured in any health insurance scheme. The previously insured are respondents who had insurance cards but was not valid for them to access healthcare with at the time of the survey because they did not renew their membership in the year 2012, whiles the never insured are those who have never enrolled in any health insurance before. Respondents’ socio-demographic characteristics were also captured by asking them to state their age, sex, marital status, religion, level of complete education, size of household, rural or urban nature of locality, employment status, self-assessed health status, and weekly consumption expenditure (S1 Appendix).

### Ethical considerations

Ethical clearance for the research was obtained from the Ghana Health Service (GHS) Ethical Review Committee (ERC) with ethical clearance number: GHS-ERC: 08/5/11. The ethical clearance approved for informed consent to be obtained from the household heads. Literate respondents provided written informed consent, while illiterate respondents thumb-printed the informed consent form read to them in their local language before participating in the study. Data was also analysed anonymously.

### Statistical analysis

All statistical analysis was performed with Stata software version 12.1. First, the averages of the respondents’ demographic and socio-economic statistics were calculated. The differences in these descriptive statistics between the insured and uninsured, and also between the previously insured and never insured, was done using a two-sample independent t-test.

Two-sample independent t-tests were then used to test the hypothesis that there is no difference in the perception on healthcare quality between the insured and uninsured; or between the previously insured and never insured. Given the ordinal five-level categories of the dependent variables, ordinal logistic regression (proportional odds/parallel-lines model) would have been a simple straightforward, intuitive, and easy to interpret method to estimate the effect of insurance status on perception of non-technical quality of healthcare. However, the main problem with the parallel-lines model is that the proportional odds assumption is often violated. To ascertain this, we used the *brant command* in Stata, which does both a global test of whether the independent variables violate the proportional odds assumptions as well as a test of whether any of the independent variables separately violate the assumptions. We obtained significant test statistics for both the model estimation as well as some of the independent variables, an indication that the proportional odds assumptions of the model had been violated. To address this problem, we employed the less restrictive generalised ordered logit model (partial proportional odds model), which relaxes the proportional odds assumption and allows the effect of the independent variables to vary with the point at which the categories of the dependent variable are dichotomised. The standard formula for the predicted probability in the partial proportional odds model is denoted as:

$$P\left(Y_{i}<j\right)=\frac{\exp(\alpha_{j}X1_{i}\beta 1+X2_{i}\beta 2+X3_{i}\beta 3_{j}}{1+\left\{exp\left(\alpha_{j}X1_{i}\beta 1+X2_{i}\beta 2+X3_{i}\beta 3_{j}\right)\right\}},j=1,2,....,M-1$$ (1)

where *Y* is the ordinal dependent variable and M is the number of categories of the ordinal dependent variable [43]. The regression estimation was done for the seven indicators of healthcare quality and the overall quality index as well as controlling for key demographic and socio-economic variables [20].

The key independent variable (explanatory variable) of interest in the regression estimation is health insurance status. This variable was coded with a dichotomous response, equal to 1 if the respondent is currently insured, and 0 if currently uninsured, or equal to 1 if the respondent was never insured, and 0 if previously insured. The demographic variables used in the regression estimation as controls were age (continuous), gender (male = 0, female = 1), married or not (not married = 0, married = 1), religion, (Other religion = 0, Christian = 1), residential zone (rural = 0, urban = 1), number of people in the household (continuous), and number of health facility visits (continuous). Other socio-economic variables included as controls were level of completed education (below primary level = 0, primary education and above = 1) and employment status (unemployed = 0, employed = 1).

## Results

### Descriptive characteristics and health insurance enrolment

Of the 1,920 households sampled, the baseline survey interviewed 1,903 household heads (948 in the Western region and 955 in the Greater Accra region), representing a 99.1% interview consent rate; 17 household heads declined to participate in the survey.

The descriptive characteristics of respondents and the differences in these characteristics between the currently insured and currently uninsured and between the previously insured and never insured are presented in Table 1. Approximately 40% of the respondents were enrolled in health insurance at the time of the survey and of the uninsured, approximately 70% had never been insured in any health insurance scheme.

**Table 1:** Descriptive characteristics and health insurance enrolment of respondents.

		Total Obs.	Mean	Std Error	Currently Insured	Currently Uninsured	Diff.	p-value (t-test)	Previously Insured	Never Insured	Diff.	p-value (t-test)
**A. Demographics**	Average Age (Years)	1903	44.6	0.53	48.4	42.2	6.2	0.000	45.3	40.9	4.4	0.000
	% Females	1903	36.4	1.35	37.1	35.9	1.1	0.619	45.7	31.7	14.0	0.000
	% Married	1900	52.9	1.47	56.1	50.8	5.3	0.032	50.4	50.9	0.5	0.887
	% Christian	1903	88.6	1.03	90.9	87.0	3.9	0.010	90.2	85.7	4.6	0.019
	% Living in Rural Communities	1903	50.2	6.33	50.2	50.2	0.0	0.994	45.7	52.1	-6.4	0.136
	Average Household size	1903	3.7	0.07	3.9	3.7	0.2	0.070	3.8	3.6	0.2	0.149
**B. Socio-economic**	% Primary education and above	1895	73.2	1.64	76.6	70.9	5.6	0.013	70.9	70.9	0.0	0.996
	% Employed	1903	86.3	1.10	83.3	88.3	-5.0	0.011	86.8	88.9	2.1	0.268
	Average Annual Household Expense	1867	3685.4	393.4	3432.7	3847.3	-414.6	0.450	3103.2	4169.6	1066.4	0.216
**C. Health**	% Good health status	1885	84.6	1.09	78.7	88.5	-9.7	0.000	87.2	88.9	1.7	0.411
	Average number of Illness	1903	1.1	0.04	1.3	0.9	0.4	0.000	0.9	0.9	0.0	0.290
	Average number of health facility visits	1812	0.8	0.06	1.1	0.5	0.6	0.000	0.6	0.5	0.1	0.000
**D. Insurance**	% Currently Insured	1903	39.6	1.6								
	% Never Insured	1903	69.7	1.8								

Source: COHEiSION Project baseline survey (March 2012), N = 1,903 household heads. Note: Standard errors are robust and corrected for clustering at the health facility level.

*: p<0.10

**: p<0.05

***: p<0.01

The average age of respondents was approximately 45 years old with 36.4% being female. Slightly more than half (52.9%) were married with the majority of them (88.6%) being Christian. About half (50.2%) of the respondents lived in rural communities with an average household size of approximately four people. The currently insured were older (48 years), with slightly more female (37%) and more married (56.1%) respondents, equally likely to live in rural (50.2%) or urban communities, and more likely to have a household close to the average size (3.9) compared to the currently uninsured. These differences in the demographic characteristics between the currently insured and currently uninsured were statistically significant at the 95% confidence level except for the percentage of females and the percentage living in rural communities. Similarly, the previously insured were significantly more likely to have higher averages and proportions in these demographic characteristics than the never insured, except for the married percentage and percentage living in rural communities (See Panel A of Table 1).

Most (73.2%) of the respondents completed a primary or above level of education with the majority (86.3%) gainfully employed. The average annual household consumption expenditure was GH₵3,685.40 ($2,538.61) in 2012. Although the currently insured (76.6%) were significantly more likely to have completed a primary or above level of education, the currently uninsured (88.3%) were more likely to be gainfully employed and also more likely to spend more (GH₵3,847.30 or $2,650.13) on household consumption. Except for the annual household consumption expenditure, these differences between the currently insured and currently uninsured were statistically significant at the 95% confidence level. The previously insured, however, were less likely to have higher averages and proportions in these socio-economic characteristics than the never insured, except for the percentage with primary or above level of education where both the previously insured and never insured were equal (see Panel B of Table 1).

A high proportion (84.6%) of respondents indicated that they were in good health; in the six months prior to the household survey, the average number of illnesses was 1.1 and the average number of health facility visits was 0.8. The currently insured (78.7%) were significantly less likely to report being in good health; they were significantly more likely (1.3 times) to report being ill and more likely (1.1 times) to visit a health facility. The previously insured (87.2%) were, however, less likely to report being in good health but equally likely (0.9 times) to report being ill and more likely (0.6 times) to visit a health facility compared to the never insured (see Panel C of Table 1).

### Perception of non-technical quality of healthcare

Generally, the average perception of healthcare quality among the respondents was relatively low, with the highest average perception approximately three on the scale (equivalent to neutral on the five-point Likert scale) for the ‘process of lodging complaints at the health facility’.

The currently uninsured perceived the quality of healthcare to be better than the currently insured on all seven quality indicators. The average perception of the currently uninsured (2.09, p-value = 0.000; 2.81, p-value = 0.018; 2.21, p-value = 0.000; 2.36, p-value = 0.000; 2.48; 2.59, p-value = 0.000; 2.08, p-value = 0.000, and 2.33, p-value = 0.000) was significantly higher than that of the currently insured on: services provided; process of lodging complaints; information provision; waiting time; availability of prescribed drugs; equal treatment of insured and uninsured patients; fair queuing system; and, overall average perception index, respectively.

Similarly, when the currently uninsured were disaggregated into the previously insured and never insured, the perceptions of the never insured (2.15, p-value = 0.000; 2.85, p-value = 0.351; 2.26, p-value = 0.005; 2.64, p-value = 0.033; 2.08, p-value = 0.824 and 2.36, p-value = 0.023) were found to be higher than the previously insured on: services provided; process of lodging complaints; information provision; waiting time; equal treatment of insured and uninsured patients; fair queuing system; and, overall average perception index, respectively.

Table 2 presents the differences in the average perceptions on healthcare quality between the currently insured and currently uninsured, and between the previously insured and never insured.

**Table 2:** Differences between the insured and uninsured in the average perception of quality of 64 health facilities.

	Total Obs.	Mean	Std. Error	Currently Insured	Currently Uninsured	Diff.	p-value (t-test)	Previously Insured	Never Insured	Diff.	p-value (t-test)
How satisfied are you with the services provided by the health facility?	1869	1.99	0.02	1.84	2.09	0.24	0.000	1.94	2.15	-0.21	0.000
How satisfied are you with the process of lodging complaint at the facility?	1866	2.78	0.02	2.73	2.81	0.08	0.018	2.78	2.85	-0.04	0.351
How satisfied are you with the information provided by the health facility?	1874	2.11	0.02	1.95	2.21	0.26	0.000	2.09	2.26	-0.16	0.005
How satisfied are you with the waiting time at the facility?	1789	2.27	0.02	2.14	2.36	0.23	0.000	2.26	2.40	-0.14	0.042
I received all prescribed drugs from the facility	1879	2.41	0.03	2.31	2.48	0.17	0.006	2.50	2.47	0.03	0.721
Health personnel treats patients with insurance cards in an equal way as patients without cards	1884	2.50	0.03	2.24	2.59	0.22	0.000	2.46	2.64	-0.18	0.033
There is a fair queuing system at the health facility.	1884	2.01	0.02	1.90	2.08	0.18	0.000	2.07	2.08	-0.01	0.824
Overall Average Perception	1886	2.26	0.02	2.14	2.33	0.19	0.000	2.25	2.36	-0.11	0.023

Source: COHEiSION Project baseline survey (March 2012), N = 1,903 household heads. Note: Standard errors are robust and corrected for clustering at the health facility level.

*: p<0.10

**: p<0.05

***: p<0.01

However, for the perception on availability of prescribed drugs at the health facility (2.5, p-value = 0.721), the perceptions of the previously insured were higher than that of the never insured, but the difference was statistically insignificant at the 95% confidence level.

### Effect of current insurance status on perceived quality of healthcare

Table 3 presents the results of the generalised logit regression estimation of the effect of current health insurance status on the perception of healthcare quality. Only the odds ratio of the independent variable of interest (insurance status) and the constant are reported for the various ratings of quality perceptions. The entire results is however presented in S2 Appendix. The currently insured were significantly less likely than the currently uninsured to choose strongly disagree or very dissatisfied for the average perceptions (OR = -O.264) on healthcare quality. Similarly, the currently insured were significantly less likely to report strongly disagree or very dissatisfied (OR = -0.467; -0.294; -0.485; -0.451; -0.150; -0.346 & -0.285) with the seven indicators (perceptions on service provision, complaint lodging, information provision, waiting time, prescribed drugs, equal treatment of insured and uninsured, and queuing system). This can be seen from the negative currently insured coefficients for the strongly disagree and very dissatisfied panel in Table 3.

**Table 3:** Effect of currently insured status on perceived quality of healthcare.

		Average Perception	Service Provision	Complaint Lodging	Information Provision	Waiting Time	Prescribed Drugs	Equal Treatment	Queuing System
**Strongly Disagree or Very Dissatisfied**	Insured	-0.264*	-0.467***	-0.294**	-0.485***	-0.451***	**-0.150**	-0.346***	-0.285**
		(0.154)	(0.122)	(0.117)	(0.124)	(0.128)	(0.107)	(0.121)	(0.112)
	_cons	1.950***	0.708***	2.569***	1.341***	1.346***	1.376***	1.047***	0.823***
		(0.262)	(0.262)	(0.408)	(0.275)	(0.269)	(0.292)	(0.274)	(0.258)
**Disagree or Dissatisfied**	Insured	-0.705***	-0.731***	-0.294**	-0.755***	-0.531***	-0.451***	-0.445***	-0.518***
		(0.131)	(0.145)	(0.117)	(0.140)	(0.112)	(0.108)	(0.105)	(0.124)
	_cons	-0.103	-0.800***	1.751***	-0.408	0.132	0.395	0.116	-0.529**
		(0.272)	(0.252)	(0.396)	(0.258)	(0.262)	(0.277)	(0.272)	(0.269)
**Neutral**	Insured	**-0.088**	**-0.136**	-0.294**	**-0.248**	**-0.173**	**-0.100**	**-0.172**	**0.024**
		(0.190)	(0.186)	(0.117)	(0.176)	(0.145)	(0.111)	(0.124)	(0.175)
	_cons	-2.495***	-2.299***	-2.488***	-2.289***	-1.683***	-0.697**	-1.376***	-1.960***
		(0.315)	(0.298)	(0.364)	(0.327)	(0.268)	(0.308)	(0.294)	(0.302)
**Agree or Satisfied**	Insured						**-0.069**	**-0.262**	**-0.118**
							(0.166)	(0.161)	(0.282)
	_cons						-1.747***	-2.147***	-2.547***
							(0.309)	(0.320)	(0.329)
	**No. of Obs.**	**1785**	**1768**	**1765**	**1774**	**1694**	**1778**	**1783**	**1783**

Source: COHEiSION Project baseline survey (March 2012), N = 1,903 household heads. Note: Standard errors in parenthesis are robust and corrected for clustering at the health facility level.

*: p<0.10

**: p<0.05

***: p<0.01.

The currently insured were also less likely than the currently uninsured to choose disagree or dissatisfied for their average perception (OR = -0.705) on healthcare quality (0R = -0.731, -0.294, -0.755, -0.531, -0.451, -0.445 & -0.518) for the seven indicators (service provision, complaint lodging, information provision, waiting time, prescribed drugs, equal treatment, and queuing system) of quality of healthcare (see the negative insured coefficients in the disagree and dissatisfied panel of Table 3).

Although the currently insured were less likely than the currently uninsured to choose neutral for their average perception or for the seven indicators of healthcare quality, it was only the results of their perception on complaint lodging (OR = -0.294) that was statistically significant (see the negative insured coefficients in the neutral panel of Table 3). The currently insured were, however, found to be less likely than the currently uninsured to choose agree or satisfied with the statements about prescribed drugs (OR = -0.069), equal treatment (OR = -0.262), and queuing system (OR = -0.118). These findings were statistically insignificant. However, In terms of the magnitude, the effect of insurance status was less extreme at the lower levels of the perceived healthcare quality categories. Thus, as one moves from disagree or dissatisfied to strongly disagree or very dissatisfied, the likelihood of the currently insured being in that category increases in magnitude.

We also found that married respondents were less likely to choose strongly disagree, very dissatisfied, disagree or dissatisfied, whereas those who made health facility visits and those with primary level education or above were significantly more likely to report strongly disagree, very dissatisfied, disagree, and dissatisfied with quality of healthcare.

### Effect of previous insurance status on perceived quality of healthcare

Table 4 presents the generalised ordered logit regression estimation of the effect of never insured status on the perception on healthcare quality. Again only the odds ratio of the independent variable of interest and the constant are reported for the various ratings of quality perceptions and the entire results presented in S3 Appendix. The results show that although the never insured are more likely to choose strongly disagree or very dissatisfied and disagree or dissatisfied for their average perception (OR = 0.245) healthcare quality, this finding was statistically insignificant. The never insured were, however, significantly more likely than the previously insured to choose strongly disagree or very dissatisfied for their perception of service provision (OR = 0.330), information provision (OR = 0.294), and equal treatment (OR = 0.418) (see the positive never insured coefficients in the strongly agree or strongly dissatisfied panel in Table 4).

**Table 4:** Association of never insured status on perceived quality of healthcare.

		Average Perception	Service Provision	Complaint Lodging	Information Provision	Waiting Time	Prescribed Drugs	Equal Treatment	Queuing System
**Strongly Disagree or Very Dissatisfied**	**Never insured**	**0.245**	0.330**	**0.026**	0.294*	**0.215**	**0.078**	0.418**	**0.038**
		(0.153)	(0.164)	(0.174)	(0.157)	(0.150)	(0.153)	(0.171)	(0.143)
	_cons	1.919***	0.312	3.032***	1.183***	1.664***	1.106**	0.385	1.030***
		(0.448)	(0.387)	(0.546)	(0.428)	(0.417)	(0.463)	(0.407)	(0.386)
**Disagree or Dissatisfied**	**Never insured**	**0.245**	0.709***	**0.026**	0.556***	**0.215**	**0.129**	0.451***	0.319*
		(0.153)	(0.174)	(0.174)	(0.142)	(0.150)	(0.156)	(0.161)	(0.182)
	_cons	-0.173	-1.485***	2.040***	-0.730*	0.362	0.177	-0.384	-0.652
		(0.474)	(0.414)	(0.543)	(0.392)	(0.420)	(0.413)	(0.406)	(0.407)
**Neutral**	**Never insured**	**0.245**	**0.132**	**0.026**	**-0.159**	**0.215**	-0.277*	**0.060**	**-0.370**
		(0.153)	(0.219)	(0.174)	(0.234)	(0.150)	(0.159)	(0.174)	(0.248)
	_cons	-2.569***	-2.688***	-1.871***	-2.134***	-1.605***	-0.295	-1.793***	-1.620***
		(0.499)	(0.476)	(0.514)	(0.498)	(0.433)	(0.414)	(0.436)	(0.444)
**Agree or Satisfied**	**Never insured**						-0.633***	**-0.002**	**-0.586**
							(0.230)	(0.230)	(0.383)
	_cons						-0.618	-2.308***	-2.348***
							(0.594)	(0.520)	(0.575)
	**No. of Obs.**	**1085**	**1074**	**1070**	**1080**	**1036**	**1079**	**1084**	**1083**

Source: COHEiSION Project baseline survey (March 2012), N = 1,903 household heads. Note: Standard errors in parenthesis are robust and corrected for clustering at the health facility level.

*: p<0.10

**: p<0.05

***: p<0.01

The never insured were also more likely than the previously insured to choose disagree or dissatisfied for perceptions on service provision (OR = 0.709), information provision (OR = 0.556), equal treatment (OR = 0.451), and queuing system (OR = 0.319) (see the positive never insured coefficients in the disagree or dissatisfied panel in Table 4). In terms of magnitude, the never insured effect was more extreme at lower levels of the perceived healthcare quality ratings. This means that as one moves from disagree or dissatisfied to strongly disagree or very dissatisfied, the likelihood of the never insured being in that category reduces in magnitude.

The never insured were, however, found to be more likely than the previously insured to choose neutral for the average perception, service provision, complaint lodging, waiting time, and equal treatment, but less likely than the previously insured to choose neutral for their perceptions of information provision and prescribed drugs. However, these findings were statistically insignificant (see the positive and negative never insured coefficients in the neutral panel in Table 4). The never insured were significantly less likely to choose agreed or satisfied for only their perception of prescribed drugs (OR = 0.633), as shown in the negative never insured coefficient of the agree or satisfied panel in Table 4.

## Discussion

### Perception of non-technical quality of healthcare

We found that within the NHIS in Ghana, there were significant differences between the currently insured and currently uninsured; and between the previously insured and never insured for most of the seven indicators of perceived quality of healthcare. Although the perceptions of both the currently insured and currently uninsured were generally low, the perceptions of the currently insured were significantly lower than that of the currently uninsured. However, the perceptions of the never insured were significantly better than the previously insured. The differences in the service delivery processes at the health facility for the insured and uninsured might be the reason for this difference in the perceived quality of healthcare between the currently insured and currently uninsured and between the never insured and previously insured. This difference in the service delivery process is a consequence of the introduction of health insurance in Ghana. Thus, the experiences of the insured at the healthcare facilities have a negative influence on their perceptions of healthcare quality compared to the uninsured. This finding is consistent with findings by Fenenga et al. [18] that insured clients in Ghana expressed dissatisfaction with long waiting times, inadequate information provision, unfair queuing systems, poor staff attitudes, and poor quality of drugs at accredited health facilities. Clearly, dissatisfaction with the care delivery processes for insured clients have a negative effect on their perceptions of the quality of healthcare.

### Effect of insurance status on perception of healthcare quality

Health insurance enrolment had a significant negative effect on the perception on healthcare quality. The regression estimation indicates that being currently insured is associated with a significantly lower perception on healthcare quality. When the currently uninsured were disaggregated into never insured and previously insured, being never insured was associated with a significantly higher perception of most of the healthcare quality indicators. This means that once people are insured, they tend to perceive the quality of healthcare to be poor compared to those uninsured, whereas those who have never been insured before tend to perceive the quality of healthcare as better than those who once had health insurance and dropped it. This finding is, however, in contrast with that of Robyn et al. [20] who found that both the insured and uninsured in the Nouna District of Burkina Faso gave high ratings for quality of healthcare delivery.

A plausible explanation for this could be that under the NHIS, insured clients actually receive lower levels of actual quality due to the differences in the healthcare delivery process for the insured and uninsured at the health facilities. The service delivery processes for NHIS accredited health providers are such that they have to process claims forms for consultation, diagnosis, and drugs for insured clients. However, for the uninsured, providers only have to provide care and collect cash. Therefore, the uninsured are kept in a different queue, quickly seen at the out-patients’ department for consultation and at the pharmacy for drugs, whereas the insured spend long hours in queues to go through the NHIS process for receiving healthcare. The uninsured, therefore, do not experience the long waiting times that the insured experience. This coupled with the long delays, in excess of over six months, in NHIS reimbursement, creates a situation where healthcare providers have no incentive to invest in the provision of quality services for insured clients when they can provide services to uninsured clients and receive cash immediately. This translates into the uninsured being satisfied with the waiting times, and the insured feeling they spend long hours in queues at the health facilities, as found in this study, and also documented by Fenenga et al. [18].

The NHIS purchasing contracts with accredited providers specifies the prescription of generic drugs for insured clients. Thus, providers who dispense branded drugs are not reimbursed. This, in addition to the delays in the reimbursement of claims, serves as a further disincentive for providers to stock generic drugs. Insured clients are asked to buy generic drugs from outside the facility, whilst uninsured clients pay cash for branded drugs at prevailing market prices at the health facilities. Insured clients interpret their prescriptions of generic drugs and branded drugs for the uninsured as discriminatory, as documented by Jehu-Appiah et al. [33] and Fenenga et al. [18]. These differences in the healthcare delivery process for the insured and uninsured might represent a lower level of actual quality to insured clients, which translates into their negative perceptions of healthcare quality as found in this study.

## Conclusion

This study demonstrated that health insurance status matters in the perception of the non-technical quality of healthcare in Ghana. The findings suggest that people’s perceptions of healthcare quality may be shaped by their actual experiences at the health facilities, and these experiences differ depending on their insurance status. The insured who experience long delays and are served with generic drugs due to the different services delivery process they go through perceive the quality of care they receive to be low whiles the uninsured who pay cash for branded drugs and spend less time perceive the quality of the care they receive to be relatively higher. The implication is that enrolment levels in the NHIS are low because service quality is perceived to be lower for people with health insurance compared to those who make out-of-pocket payments. The insured who are dissatisfied with the quality of care may drop out because they think they are better off opting for out-of-pocket payments, while others may renew because they believe the financial protection of not having to make out-of-pocket payments over-rides the low quality of care. Therefore, it is important that policy makers consider redesigning, reorganising, and reengineering the NHIS, particularly the provider payment mechanism, to ensure the provision of quality healthcare services for all.

The authors acknowledge some limitations that might have affected the results. The sample for the study was drawn from two of the 10 regions in Ghana. The sample size might therefore not be representative of the Ghanaian population. The survey questionnaire was translated from English to the two local languages (Ga and Fante) of the study regions for the respondents in the rural communities to understand and thus could be affected by interviewer bias. The perception questions could lose their actual meaning in the process. The perceptions of respondents may differ significantly in the other regions of the country due to differences in the number of public and private health facilities in these other regions. The two groups (insured and uninsured) were significantly different in terms of age, marital status, religion, educational level, employment status, and health status, which were controlled for in the analysis. It is possible that these two groups also differ on other unobserved factors, which could not be controlled for in the analysis, and therefore, the observed effect of insurance status on perceived quality of healthcare could be the result of systematic differences between the two groups or other unobserved factors. These limitations notwithstanding, the findings from this study remain relevant to the NHIS, the Ghana Health Service, and other countries implementing social health insurance schemes for the provision of quality healthcare services to the insured and uninsured and the sustainability of such schemes.

## Supporting information

S1 Appendix Baseline survey questionnaire. (DOC)

S2 Appendix Effect of currently insured status on perceived quality of healthcare. (DOCX)

S3 Appendix Association of never insured status on perceived quality of healthcare. (DOCX)

S4 Appendix Perceived quality data. (DTA)

## Abbreviations

AIGHD: Amsterdam Institute for Global Health and Development
CBHI: Community-based Health Insurance
CBI: Community-based Insurance
CHAG: Christian Health Association of Ghana
COHEiSION: Client Oriented Health Insurance System in Our Nation
G-DRG: Ghana Diagnostic Related Groupings
GHS: Ghana Health Service
MOH: Ministry of Health
NHIA: National Health Insurance Authority
NHIF: National Health Insurance Fund
NHIS: National Health Insurance Scheme
NOW: Netherlands Organization for Scientific Research
SSA: Sub-Saharan Africa
SSNIT: Social Security and National Insurance Trust
VAT: Value Added Tax
VU: Vrije University, Amsterdam
WOTRO: Science for Global Developmen

## References

[1] World Bank. World development indicators Washington DC: World Bank Group 2008; Available: www.worldbank.org/sites/default/files/wdi08.pdf. Accessed 24 August 2011.

[2] Gottret P, Schieber G. Health financing revisited: a practitioner’s guide Washington DC: World Bank 2006 Available: www.siteresources.worldbank.org/INTHSD/Resources/topics/Health-Financing/HFRFull.pdf. Accessed 12 June 2012.

[3] Atim C, Fleisher LK, Hatt L, Musau S, Arur A. Health financing in Africa today: challenges and opportunities. Africa’s health in 2010. Washington, DC: Academy for educational development and Bethesda, MD: Health Systems; 2008: 20: 20; Available: www.hhaonline.org/hso/system/files/Health_Financing_in_Africa_Today_FIN[1].pdf. Accessed 12 June 2012.

[4] Xu K, Evans DB, Kawabata K, Zeramdini R, Klavus J, Murray CJL. Household catastrophic health expenditure: A multicounty analysis. Lancet. 2003; 362: 111–117. doi: 10.1016/S0140-6736(03)13861-5 12867110 10.1016/S0140-6736(03)13861-5

[5] Basaza R, Criel B, Van der Stuyft P. Community health insurance in Uganda: why does enrolment remain low? A view from beneath. Health Policy. 2008; 87: 172–184. doi: 10.1016/j.healthpol.2007.12.008 18280608 10.1016/j.healthpol.2007.12.008

[6] Missoni E, Solimano G. Towards universal health coverage: the Chilean experience. Geneva Switzerland: World Health Organization. Background paper No. 4; 2010. Available: www.who.int/healthsystems/topics/financing/healthreport/4Chile.pdf. Accessed 12 May 2012.

[7] Durairaj V, D’Almeida S, Kirigia J. Ghana’s approach to social health protection. World Health Report 2010. Geneva: World Health Organization; Background paper No.2; 2010. Available: www.who.int/healthsystems/topics/financing/healthreport/GhanaNo2Final.pdf. Accessed 24 August 2011

[8] Franco LM, Diop FP, Burgert CR, Gamble K, Kelly AG, Makinen M, et al Effects of mutual health organizations on use of priority health care services in urban and rural Mali: a case-control study. Bull World Health Org. 2008 86: 830–838. doi: 10.2471/BLT.08.051045 19030688 10.2471/BLT.08.051045 PMC2649553

[9] World Health Organization. The world health report: health systems financing; the path to universal coverage Geneva: World Health Organization; 2010 Available: www.who.int/whr/2010. Accessed 21 November 2011. 10.2471/BLT.10.078741 PMC2878164 20539847

[10] McIntyre D, Garshong B, Mtei G, Meheus F, Thiede M, Akazlie J, et al Beyond fragmentation and towards universal coverage: Insights from Ghana, South Africa and The United Republic of Tanzania. Bull World Health Org. 2008; 86 (11): 817–908. 10.2471/BLT.08.053413 PMC2649570 19030693

[11] National Health Insurance Authority. National Health Insurance Authority 2011 Annual Report. Accra: NHIA; 2012.

[12] National Health Insurance Authority. National Health Insurance Authority 2013 Annual Report. Accra: NHIA; 2014.

[13] De Allegri M, Kouyate B, Becher H, Gbangou A, Pokhrel S, Sanon M, et al Understanding enrolment in community health insurance in Sub-Saharan Africa: A population-based case-control study in rural Burkina Faso. Bull World Health Org. 2006; 84: 852–858. 17143458 10.2471/blt.06.031336 PMC2627536

[14] Jehu-Appiah C, Aryeetey G, Agyepong I, Spaan E, Baltussen R. Household perceptions and their implications for enrolment in the national health insurance scheme in Ghana. Health Policy Plan. 2012; 27(3): 222–233. doi: 10.1093/heapol/czr032 21504981 10.1093/heapol/czr032

[15] Criel B, Waelkens MP. Declining subscriptions to the Maliando mutual health organization in Guinea-Conakry (West Africa): What is going wrong? Soc Sci Med. 2003; 57: 1205–19. 12899905 10.1016/s0277-9536(02)00495-1

[16] Dong H, De Allegri M, Gnawali D, Souares A, Sauerborn R. Drop-out analysis of community-based insurance membership at Nouna, Burkina Faso. Health Policy. 2009; 92: 174–9. doi: 10.1016/j.healthpol.2009.03.013 19394105 10.1016/j.healthpol.2009.03.013

[17] Spaan E, Mathijssen J, Tromp N, McBain F, Have AT, Baltussen R. The impact of health insurance in Africa and Asia: A systematic review. Bull World Health Org. 2012; 90: 685–692. doi: 10.2471/BLT.12.102301 22984313 10.2471/BLT.12.102301 PMC3442382

[18] Fenenga CJ, Boahene K, Arhinful D, Tobias RW, Inge H. Do prevailing theories sufficiently explain perceptions and health seeking behavior of Ghanaians? Int J Health Plann Manage. 2014; 29(1): 26–42. doi: 10.1002/hpm.2159 23303726 10.1002/hpm.2159

[19] Bruce E, Narh-Bana S, Agyepong I. Community satisfaction, equity in coverage and implications for sustainability of the Dangme West Health Insurance Scheme Project No. 2001/GD/08 technical report series No. 9. Accra: Ghanaian Dutch Collaboration for Health Research and Development; 2008.

[20] Robyn PJ, Barnighausen T, Souares A, Savadog G, Bicaba B, Sie A, et al Does enrolment status in community-based insurance lead to poorer quality of care? Evidence from Burkina Faso. Int J Equity Health. 2013; 12:31–44. doi: 10.1186/1475-9276-12-31 23680066 10.1186/1475-9276-12-31 PMC3665463

[21] Sofaer S, Firminger K. Patient perceptions of the quality of health services. Annu Rev Public Health. 2005; 26: 513–559. doi: 10.1146/annurev.publhealth.25.050503.153958 15760300 10.1146/annurev.publhealth.25.050503.153958

[22] Marshall GN, Hays RD, Mazel R. Health status and satisfaction with health care: results from the medical outcomes study. J Consult Clin Psychol. 1996; 64: 380–390. 8871422 10.1037//0022-006x.64.2.380

[23] Strasser S, Ahorony L, Greenberger D. The patient satisfaction process: Moving towards a comprehensive model. Med Care Res Rev. 1993; 50: 219–248 10.1177/107755879305000205 10127084

[24] Mensah J, Oppong J, Bobi-Barimah K, Frimpong G, Sabi W. An evaluation of the Ghana National Health Insurance Scheme in the context of the health MDGs. Working paper No. 40, Global development network 1999–2009, working paper series. 2010. Available: www.rwi-essen.de/media/content/pages/publikationen/ruhr-economic-papers/REP_09_157.pdf. Accessed 14 July 2012.

[25] Schieber G, Cashin C, Saleh K. Health financing in Ghana Washington DC: The World Bank; 2012 Available: www.jointlearningnetwork.org/uploads/files/resources. Accessed 14 July 2013.

[26] Basu S, Andrews J, Kishore S, Panjabi R, Stuckler D. Comparative Performance of Private and Public Health Systems in Low- and Middle- Income Countries: A Systematic Review. PloS Med. 9(6): e1001244 doi: 10.1371/journal.pmed.1001244 22723748 10.1371/journal.pmed.1001244 PMC3378609

[27] Arhinful DK. The solidarity of self-Interest: Social and cultural feasibility of rural health insurance in Ghana. Leiden: African studies canter research report; 2003. Available: www.openaccess.leidenuniv.nl. Accessed 20 August 2011.

[28] NHIA. National health insurance authority 2008 Annual report NHIA Accra, Ghana; 2009

[29] Nguyen HT, Rajkotia Y, Wang H. The financial protection effect of Ghana national health insurance scheme: Evidence from a study of two rural districts. Int J Equity Health. 2011; 10: 4–16. doi: 10.1186/1475-9276-10-4 21247436 10.1186/1475-9276-10-4 PMC3031235

[30] Agyepong IA, Adjei S. Public social policy development and implementation: A case study of the Ghana national health insurance scheme. Health Policy Plan. 2008; 23(2): 150–160. doi: 10.1093/heapol/czn002 18245803 10.1093/heapol/czn002

[31] Gronroos C. A service quality model and its marketing implications. Eur J Mark. 1984; 18(4): 36–64.

[32] D’souza SC, Sequeira AH. Measuring the Customer-Perceived Service Quality in Health Care Organization: A Case Study. J Health Manag. 2012; 14(1): 27–41.

[33] Jehu-Appiah C, Aryeetey G, Spaan E, De Hoop T, Agyepong I, Baltussen R. Equity aspects of the national health insurance scheme in Ghana: Who is enrolling, who is not and why? Soc Sci Med. 2011; 72: 157–165. doi: 10.1016/j.socscimed.2010.10.025 21145152 10.1016/j.socscimed.2010.10.025

[34] Boateng D, Awunyor-Vitor D. Health insurance in Ghana: Evaluation of policy holders’ perception and factors influencing policy renewal in the Volta Region. Int J Equity Health. 2013; 12: 50–60. doi: 10.1186/1475-9276-12-50 23822579 10.1186/1475-9276-12-50 PMC3716631

[35] Alderman H, Lavy V. Household responses to public health services: Cost and quality trade-offs. World Bank Res Obs. 1996; 11(1): 3–22.

[36] Lavy V, Germain JM. Quality and cost in health care choice in developing countries. Living standards measurement study (LSMS) working paper No. LSM 105. Washington DC: The World Bank. 1994.

[37] Lavy V, Quigley JM. On the economics of medical care: The choice of quality and intensity of medical care in Ghana Washington DC, London: The World Bank, Sage, 1991; 627–685.

[38] Klemick H, Leonard KL, Masatu MC. Defining access to health care: Evidence on the importance of quality and distance in rural Tanzania. Am J Agric Econ. 2009; 91(2): 347–358.

[39] Sahn DE, Younger SD, Genicot G. The demand for health care services in rural Tanzania. Oxf Bull Econ Stat. 2003; 65(2): 241–26.

[40] Andaleeb SS. Service quality perceptions and patient satisfaction: a study of hospitals in a developing country. Soc Sci Med. 2001; 52: 1359–1370. 11286361 10.1016/s0277-9536(00)00235-5

[41] Akin JS, Hutchinson P. Health-care facility choice and the phenomenon of bypassing. Health Policy Plan. 1999; 14(2): 135–151. 10538717 10.1093/heapol/14.2.135

[42] GSS. 2010 Population and Housing Census. Ghana Statistical Service Accra, Ghana 2011 Available: http://www.statsghana.gov.gh/docfiles/2010phc/Census2010_Summary_report_of_final_results.pdf. Accessed 14 July 2013.

[43] William R. Generalized ordered logit/partial proportional odds model for ordinal dependent variables. Stata J. 2006; 6(1): 58–82.

