# Supplementary material for: Perceptions of healthcare quality in Ghana: Does health insurance status matter?
Source: PLoS One. 2018 Jan 16;13(1):e0190911. doi: 10.1371/journal.pone.0190911 (PMC5770037; doi:10.1371/journal.pone.0190911)
Supplement: S1 Appendix — (DOC) [file pone.0190911.s001.doc]

| **SECTION A: INTRODUCTION AND INFORMED CONSENT** |
| --- |
| ***Hello. My name is […………………………………………………………………...].***  ***I’m working on a health research project titled “Towards a client oriented health insurance system in Ghana”. The project is being conducted by the University of Ghana, Legon.***  ***The purpose of this survey is to measure how clients perceive the healthcare services and health insurance services and to learn about how these services can be offered in a more client oriented manner. Gaining insight into what people experience and perceive will help to improve health care access and quality. Your cooperation in this survey will contribute to reaching this goal.***  ***I would like to assure you that your responses will be confidential, and will only be used for the purposes of this study. Your name will not be disclosed to anyone and the answers you give will be processed in such a way that they cannot be linked to your name.***  ***I would greatly appreciate your participation in this survey. The interview will take approximately (6o minutes) one hour to complete. You can ask any question during the interview and have the right to refuse to answer questions, or terminate the interview at any time. If you have any questions during the interview you are free to ask***  ***Are there any questions you would like to ask at this moment?***  ***Do you declare to have understood the purpose of this survey and agree to participate in this survey?***  ***YES [ ] NO [ ]***  ***NAME / SIGNATURE RESPONDENT: ………………………………………………/……………………………………….………….*** |

**S1 Appendix. Baseline Survey Questionnaire**

***Note for surveyors: if respondents want more information they can contact Dr. Daniel Arhinful, Mrs. Christine Fenenga, Mr. Stephen Duku or Noguchi Memorial Institute for Medical Research, University of Ghana P.O. Box LG 581, Legon, Accra, Ghana Tel (Office) 233-24 3613635.***

| **COHEiSION PROJECT GHANA BASELINE SURVEY**  **SECTION A: IDENTIFICATION SHEET** | | | | | | | | |
| --- | --- | --- | --- | --- | --- | --- | --- | --- |
| 1. HOUSEHOLD NUMBER: | | 2a. NAME OF HOUSEHOLD HEAD: ……………………………………………………………………………….  2b. CALL NAME HOUSEHOLD HEAD: …………………………………………………………………………... | | | | | | |
| 3a.Address of Respondent: ………………………………………………. ………………………………………………………………………………………  3b.Detail description of respondent house:………………………………….……………………………………………………………………………………  4a. LOCALITY: ………………………………………………….. 4b.EA NUMBER  5a. DISTRICT……………………………………………………..…… 5b. District code: | | | | | | | | |
| Start Time: | |  | | | | | | |
| 6. Date (dd/mm/yy) | | i) First visit | | ii) Second visit: | | | | iii) Third visit: |
| 7. Result: | | Interview…………………. 1  Dwelling not found…….2  Absent……………………….3  Refused (specify)……….4  Other (specify)…………..99 | | | Interview……………………1  Dwelling not found…….2  Absent……………………….3  Refused (specify)……….4  Other (specify)…………..99 | | | Interview……………………1  Dwelling not found…….2  Absent……………………….3  Refused (specify)……….4  Other (specify)…………..99 |
| 8. Name of the interviewer: | |  | | | | | | |
| 9.Code of interviewer | |  | |  | | |  | |
| Remarks: | |  | | | | | | |
| VERIFICATION *(to be filled in by supervisor)* | | | | | | | | |
| 10. Name of Supervisor:  …………………………………… | 11. Re-interview needed?  1… YES 2….NO | | 12. This Household to be replaced by:  HH number. | | | 13. This Household replaces:  HH number | | |
| Remarks:  …………………………………………………………………………………………………………… | | | | | | | | |

(*Remark: household number consist of district no (2 spaces) and household number (3 spaces)*

| **SECTION B: HOUSEHOLD ROSTER**  **RESPONDENT: HOUSEHOLD HEAD** | | | | | | | | |
| --- | --- | --- | --- | --- | --- | --- | --- | --- |
| **ID code** | **B1: Name**  Please give me the names of the persons who usually live and eat in your household.  *Please start with head of household.* | **B2: Sex**  M…1  F ….2 | **B3: What is your/[NAME]’s relationship with the household head**  Head of HH…….………..1  Spouse……..……………..2  Son/Daughter …………3  Son/Daughter in law..4  Grandchild..…………….5  Parent ……………..........6  Parent in law……………7  Bro/Sis………..…………..8  Other relative………….9  Not related…….........10  Other specify…………99 | **B4: What was your/ [NAME]’s age at last birthday?**  **If aged less than 12 months, write 0.**  **If less than 18 years, skip to B6** | **ONLY ADULTS >=18**  **B5: What is your/ [NAME]’s marital status?**  Never married….1  Married…….… …..2  Divorced…………..3  Separated…… …..4  Widow(er)…….. .5  Living together…6 | **B6: What is your/[NAME]’s religion?**  Christian…..1  Muslim …….2  Traditional..3  None ………..4  Other ……….99 | **B7: What is your / [NAME]’s highest level of completed education?**  None………..…………1  < primary …..……….2  Primary ….…………..3  Middle/JSS…..……..4  Secondary/SSS…...5  Tech/Vocational….6  Polytechnic………….7  First Degree…………8  Postgraduate……….9 | |
|  |  |  |  |  |  |  | **a. Level** | **b. Grade** |
| 01 |  |  |  |  |  |  |  |  |
| 02 |  |  |  |  |  |  |  |  |
| 03 |  |  |  |  |  |  |  |  |
| 04 |  |  |  |  |  |  |  |  |
| 05 |  |  |  |  |  |  |  |  |
| 06 |  |  |  |  |  |  |  |  |
| 07 |  |  |  |  |  |  |  |  |
| 08 |  |  |  |  |  |  |  |  |
| 09 |  |  |  |  |  |  |  |  |
| 10 |  |  |  |  |  |  |  |  |

**SECTION C: SOCIAL CAPITAL**

**GROUPS AND NETWORKS:** I would like to ask you questions about groups and associations in your community (For household members 18 years and above).

| **ID** | **C1**  Do you [NAME] belong to any organized association/group?  **Yes…….1**  **No……..2**  **If No, SKIP to C12**  *Note****:*** *organized group does exclude family or community* | **C2**  Which associations/group do you [NAME] belong to?  **Religious group………..……….1**  **Agricultural group…….…. …..2**  **Handicraft/Sewing group…..3**  **Education committee.……….4**  **Funeral society………………...5**  **Savings/Credit group…………6**  **Women’s group………………..7**  **Men group…………………….…8**  **Youth association……………..9**  **Cultural group…………………10**  **Football club…………………..11**  **Keep fit……….…..…………….12**  **Fishery association……….. .13**  **Other specify………………….99**  **RECORD 3 MOST IMPORTANT RESPONSES** | **C3**  How long have you [NAME] been a member of your most important association/group?  **No. of months……**  **No. of years………..**  *Note: Question C4 till C12 relate to this most important association/ group* | **C4**  Do you [NAME] make any contributions in this association/group?  **Registration fee….1**  **None …….…………..2**  **Annual Dues……….3**  **Labor.…………………4**  **Caretaking ………….5**  **Other specify.……99**  **RECORD 3 MOST IMPOATANT RESPONSES** | **C5**  How many members are there in this association/group?  **………….members** | **C6**  How often does this active association/group meet?  **Once a week……….1**  **Once in 2weeks…..2**  **Once a month……..3**  **Every quarter.….. ..4**  **Other specify…....99** |
| --- | --- | --- | --- | --- | --- | --- |
| **01** |  |  |  |  |  |  |
| **02** |  |  |  |  |  |  |
| **03** |  |  |  |  |  |  |
| **04** |  |  |  |  |  |  |
| **05** |  |  |  |  |  |  |
| **06** |  |  |  |  |  |  |
| **07** |  |  |  |  |  |  |
| **08** |  |  |  |  |  |  |
| **09** |  |  |  |  |  |  |
| **10** |  |  |  |  |  |  |

**SECTION C: SOCIAL CAPITAL (GROUPS AND NETWORKS).**

| **ID** | **C7**  How often do you [NAME] attend meetings?  **All the time………..1**  **Most times………..2**  **Half the times……3**  **Sometimes……….4**  **Never……………….5**  **Other specify….99** | **C8**  Are you [NAME] a leader in your associations/ group?  **Yes………..….1**  **No…………….2**  *Note: a leader can be any in an executive body* | **C9**  Who formed the association?  **By the community………1 By the Government……2 By an NGO……..………….3 Other specify…………..99** | **C10**  Why did you choose to join this association/group?  **Socializing………..…..1**  **Sharing info……….....2**  **Supporting others….3**  **Development …….. .4**  **Recommended….....5**  **Other specify.………99** | **C11**  Does this active association/group link-up with groups outside your community?  **Yes occasionally…..1**  **Yes frequently……..2**  **No...…………………….3** | **C12**  Which of the associations/ groups is the most dominant in your community?  **Religious group………..……….1**  **Agricultural group…….…. …..2**  **Handicraft/Sewing group…..3**  **Education committee.……….4**  **Funeral society………………...5**  **Savings/Credit group…………6**  **Women’s group………………..7**  **Men group…………………….…8**  **Youth association……………..9**  **Cultural group…………………10**  **Football club…………………..11**  **Keep fit……….…..…………….12**  **Fishery association……….. .13**  **Other specify………………….99**  **RECORD 3 MOST IMPORTANT RESPONSES** |
| --- | --- | --- | --- | --- | --- | --- |
| **01** |  |  |  |  |  |  |
| **02** |  |  |  |  |  |  |
| **03** |  |  |  |  |  |  |
| **04** |  |  |  |  |  |  |
| **05** |  |  |  |  |  |  |
| **06** |  |  |  |  |  |  |
| **07** |  |  |  |  |  |  |
| **08** |  |  |  |  |  |  |
| **09** |  |  |  |  |  |  |
| **10** |  |  |  |  |  |  |

| SECTION D: EMPLOYMENT STATUS (For household members above 15 years) ASK THE HEAD OF THE HOUSEHOLD FOR EVERY MEMBER OF THE HOUSEHOLD; IF THE HOUSEHOLD HEAD IS NOT AVAILABLE, ASK THE MOST INFORMED MEMBER AVAILABLE**?**  ***Main Occupation*** is economic activity where the respondent spends the most time during period indicated. | | | | | | | | | | | |
| --- | --- | --- | --- | --- | --- | --- | --- | --- | --- | --- | --- |
| ID No. | **D0**  In the past 12 months, did you / [NAME] work?  Yes…….1  No……..2  **if no, skip to D5** | **D1**: What is your [NAME] main occupation?  Farmer (crop)……….….1  Livestock breed………..2  Fisherman…..…….......3  Artisan …………….….….4  Trader………………….....5  Laborer......................6  Clerical…….................7  Managerial……………..8  Professional…………….9  Owner of business…10  Casual work……………11  Other(specify)….……99 | **D 2:** What type of employment is your/ [NAME] main occupation?  Full time………….1  Temporary….2 | **D 3:** How much did you/[name] earn from primary occupation?  **(LAST 12 months)**  **INDICATE AMOUNT IN GH CEDIS……….** | **D 4a**: What is your Secondary occupation?  None…………………………..….0  Farmer (crops)……………....1  Livestock breeder……….…..2  Fisherman………..…………….3  Artisan ……………………………4  Trader……………..……….…....5  Laborer……………….............6  Clerical……………………........7  Managerial……………….......8  Professional……………………9  Owner of business………..10  Casual work…………….…….11  Other…………………….………99  **If None, skip to D6** | **D 4b:** How much did you/ [name] earn from secondary occupation?  **(LAST 12 months )**  **INDICATE AMOUNT IN GH CEDIS………..** | **D5**.Why did you /[NAME] not work?  Housework……1  Unemployed...2  student…………3  retired………….4  disabled/not working ………5  other (Specify) ……………………99 | **D 6.** How much did you/ [name] earn as other income in terms of goods and services  **(LAST 30 DAYS)**  **INDICATE AMOUNT IN GH CEDIS…………** | | **D 7a.** Did you/ [name] receive remittance last year?  Yes……1  No….…2  **If No, end section** | **D7b**.Approximately how much did you/[name] received as remittance? |
| ID |  |  |  | **Primary** |  | **Secondary** |  | **Item** | **Amt** |  | **Amount** |
| 01 |  |  |  |  |  |  |  |  |  |  |  |
| 02 |  |  |  |  |  |  |  |  |  |  |  |
| 03 |  |  |  |  |  |  |  |  |  |  |  |
| 04 |  |  |  |  |  |  |  |  |  |  |  |
| 05 |  |  |  |  |  |  |  |  |  |  |  |
| 06 |  |  |  |  |  |  |  |  |  |  |  |
| 07 |  |  |  |  |  |  |  |  |  |  |  |
| 08 |  |  |  |  |  |  |  |  |  |  |  |
| 09 |  |  |  |  |  |  |  |  |  |  |  |
| 10 |  |  |  |  |  |  |  |  |  |  |  |

|  | **SECTION E: HEALTH STATUS AND HEALTHCARE UTILIZATION** (For all members of the household)  I would now like to ask you questions about your health status and that of your household members  RESPONDENT: ALL ADULT HOUSEHOLD MEMBERS ANSWER FOR THEMSELVES. MOST INFORMED HOUSEHOLD MEMBER ANSWERS FOR CHILDREN <18 YEARS. | | | | | | | | | | | | | | | | | |  |
| --- | --- | --- | --- | --- | --- | --- | --- | --- | --- | --- | --- | --- | --- | --- | --- | --- | --- | --- | --- |
| **ID** | **E1**  How would you rate your health during the last 30 days?  **READ OUT:**  **Very good……1**  **Good………….2**  **Fair…………….3**  **Bad…………….4**  **Very bad…….5** | **E2**  Is your health better or worse than last year?  **Much better…1**  **Better………….2**  **Same…………..3**  **Worse…………4**  **Much worse……5** | | **E3**  Is your health better or worse than that of other people of the same sex and age that you know in your community?  **Much better……………..1**  **Better……………………….2**  **Same………………………..3**  **Worse………………………4**  **Much worse……………..5** | | | | **E4**  During the past 30 days how much has your health interfered with your work?  **Not at all…………….1**  **Very little……………2**  **A bit……………………3**  **A lot……………………4**  **Completely…………5** | | | | **E5**  During the past one month how much has your health interfered with your social & recreational activities?  **Not at all……………..1**  **Very little…………….2**  **A bit…………………….3**  **A lot…………………….4**  **Completely………….5** | | | | **E6**  During the past one month how much has your health interfered with your household chores?  **Not at all…………1**  **Very little.………2**  **A bit……………….3**  **A lot……………….4**  **Completely…….5** | | |  |
| 01 |  |  | |  | | | |  | | | |  | | | |  | | |  |
| 02 |  |  | |  | | | |  | | | |  | | | |  | | |  |
| 03 |  |  | |  | | | |  | | | |  | | | |  | | |  |
| 04 |  |  | |  | | | |  | | | |  | | | |  | | |  |
| 05 |  |  | |  | | | |  | | | |  | | | |  | | |  |
| 06 |  |  | |  | | | |  | | | |  | | | |  | | |  |
| 07 |  |  | |  | | | |  | | | |  | | | |  | | |  |
| 08 |  |  | |  | | | |  | | | |  | | | |  | | |  |
| 09 |  |  | |  | | | |  | | | |  | | | |  | | |  |
| 10 |  |  | |  | | | |  | | | |  | | | |  | | |  |
| **ID** | **E7a**  During the past 6 months, how many times did you/[NAME] fall sick or were you/[NAME] injured?  **INDICATE NUMBER OF TIMES………**  **IF 0 skip to E9b** | **E7b**  The last time that you/[name] were sick or injured within the past 6 months, did you/[NAME] consult anyone?  **Yes ………1**  **No ……….2**  **IF 1 SKIP TO E9a.** | | **E8**  Why did you/[name] not consult anyone?  **Provider not available…................1**  **Distance to provide too far............................2**  **Could not afford health care costs ….....................3**  **Could not afford transport cost.........................4**  **Illness not too serious.....................5**  **Other[specify):…......99** | | **E9a**  In the past 6 months, how many times did you/[NAME] consult a health provider for an illness or injury or for other reasons such as preventive health or family planning?  **INDICATE NUMBER OF TIMES**  **IF 0, SKIP TO NEXT SECTION**. | | | | **E9b**  The last time that you/[NAME] consulted a health provider, what was the reason?  **USE CODE BOX E9b** | | | **E10**  To receive care for this [REASON] what was the first place that you/[NAME] visited?  **USE CODE E10**  **If 10 – 20, skip to E12** | | **E11**  What was the name of the health facility?  **USE CODE BOX E11** | | | **E12**  The last time you/[NAME] visited a health provider did you/he/she have to pay (out of pocket cash) for consultation, tests or laboratory services at this facility?  **Yes................1**  **No .................2**  **If No, skip to E15)** |  |
| 01 |  |  | |  | |  | | | |  | | |  | |  | | |  |  |
| 02 |  |  | |  | |  | | | |  | | |  | |  | | |  |  |
| 03 |  |  | |  | |  | | | |  | | |  | |  | | |  |  |
| 04 |  |  | |  | |  | | | |  | | |  | |  | | |  |  |
| 05 |  |  | |  | |  | | | |  | | |  | |  | | |  |  |
| 06 |  |  | |  | |  | | | |  | | |  | |  | | |  |  |
| 07 |  |  | |  | |  | | | |  | | |  | |  | | |  |  |
| 08 |  |  | |  | |  | | | |  | | |  | |  | | |  |  |
| 09 |  |  | |  | |  | | | |  | | |  | |  | | |  |  |
| 10 |  |  | |  | |  | | | |  | | |  | |  | | |  |  |
| **ID** | **E13a**  How much did you pay in total for consultation, tests and laboratory services out of pocket at this facility?  **amount in gh cedis** | **E13b**  Did you [NAME] pay for under-the-table, payment?  **Yes…….1**  **No…..…2**  **If no, skip to E16** | | **E14**  How much did you [NAME] pay in total under-the-table?  **AMOUNT IN GH CEDIS** | **E15**  Why did you [NAME] not pay for this service?  **Exempted.........1**  **Used my NHIS card…………….....2**  **Could not afford................3**  **Covered by employer…………4**  **Other specify..............99** | | **E16**  How long did you / [NAME] have to wait before you were attended to?  **INDICTE TIME IN HOURS AND MINUTES……**  **Hours : Mins** | | | | **E17**  Were you [NAME] satisfied with the waiting time at the Health facility?  **Yes……1**  **No…….2** | | | **E18**  Was there a well organized and fair queuing system at the health facility?  **Yes…………….1**  **No………………2** | **E19a**  Were there sufficiently good doctors/medical assistants at the health facility?  **Yes…………….1**  **No………………2** | | **E19b**  Did the Doctors/Medical Assistants/Nurses treat you [NAME] respectfully?  **Yes…………….1**  **No………………2** | | |
| 01 |  | |  |  |  | |  | |  | |  | | |  |  | |  | | |
| 02 |  | |  |  |  | |  | |  | |  | | |  |  | |  | | |
| 03 |  | |  |  |  | |  | |  | |  | | |  |  | |  | | |
| 04 |  | |  |  |  | |  | |  | |  | | |  |  | |  | | |
| 05 |  | |  |  |  | |  | |  | |  | | |  |  | |  | | |
| 06 |  | |  |  |  | |  | |  | |  | | |  |  | |  | | |
| 07 |  | |  |  |  | |  | |  | |  | | |  |  | |  | | |
| 08 |  | |  |  |  | |  | |  | |  | | |  |  | |  | | |
| 09 |  | |  |  |  | |  | |  | |  | | |  |  | |  | | |
| 10 |  | |  |  |  | |  | |  | |  | | |  |  | |  | | |

| **ID** | **E20**  Did you [NAME] get a prescription for drugs at this facility?  **Yes.............1**  **No..............2**  **if No skip to E25** | **E221**  Did you [NAME] receive all prescribed drugs from this facility?  **Yes, all drugs............1**  **Some drugs............2**  **No, none of the drugs……….…3**  **If 3 skip to E24a** | **E22**  Did you/ [name] pay for drug that you/he/she received at the facility during this visit?  **Yes……………. 1**  **No…………..…2**  **If no, skip to E23** | **E22b**  How much did you /[NAME] pay for drugs received at the facility?  **AMOUNT IN GH CEDIS**  **If answered, skip to E24a** | **E23**  Why did you/ [NAME] not pay for drugs you/he/she received at the facility during the last visit?  **Used NHIS card..............1**  **Could not afford.............2**  **Covered by employer.....3**  **Other, specify…………….99** | **E24a**  Did you [NAME] pay for drugs elsewhere?  **yes........1**  **No………2**  **AMOUNT IN GH CEDIS**  **If no, skip to E25** | **E24b**  How much did you [Name] pay for drugs elsewhere?  **AMOUNT IN GH CEDIS** | **E25**  How much did you/ [NAME] pay for transportation in total for this first visit, including transport for getting medicines?  **AMOUNT IN GH CEDIS** |
| --- | --- | --- | --- | --- | --- | --- | --- | --- |
| 01 |  |  |  |  |  |  |  |  |
| 02 |  |  |  |  |  |  |  |  |
| 03 |  |  |  |  |  |  |  |  |
| 04 |  |  |  |  |  |  |  |  |
| 05 |  |  |  |  |  |  |  |  |
| 06 |  |  |  |  |  |  |  |  |
| 07 |  |  |  |  |  |  |  |  |
| 08 |  |  |  |  |  |  |  |  |
| 09 |  |  |  |  |  |  |  |  |
| 10 |  |  |  |  |  |  |  |  |

| **SECOND VISIT (E.g. REFERRAL)** | | | | | | | | | | |
| --- | --- | --- | --- | --- | --- | --- | --- | --- | --- | --- |
| ID | **E26**  Were you referred or asked to come for a review the last time you consulted a health care provider**?**  **Yes…….1**  **No……..2**    **If No, skip to E46** | **E27**  What is the name of the health facility you visited for a review or referral?  **USE CODE BOX E11** | **E28**  The last time you/ [NAME] visited a health provider for a review or referral, did you/he/she have to pay (out of pocket cash) for consultation, tests or laboratory services?  **Yes................1**  **No .................2**  **If No, skip to E32** | **E29**  How much did you/ [NAME] pay in total for consultation, tests and laboratory services out of pocket at this facility?  **amount in gh cedis** | **E30**  Did you /[NAME] pay for under-the-table, payment?  **Yes…….1**  **No…..…2**  **If No, skip to E33** | **E31**  How much did you /[NAME] pay in total under-the-table?  **AMOUNT IN GH CEDIS** | **E32**  Why did you/[NAME] not pay for this service?  **Exempted.........1**  **Used my NHIS card…….............2**  **Could not afford................3**  **Covered by employer..........4**  **Other specify.............99** | **E33**  How long did you / [NAME] have to wait to see a doctor?  **INDICTE TIME IN HOURS AND MINUTES……**  **Hours : Mins** | | **E34**  Were you [NAME] satisfied with the waiting time at the Health facility?  **Yes……1**  **No…….2** |
| 01 |  |  |  |  |  |  |  |  |  |  |
| 02 |  |  |  |  |  |  |  |  |  |  |
| 03 |  |  |  |  |  |  |  |  |  |  |
| 04 |  |  |  |  |  |  |  |  |  |  |
| 05 |  |  |  |  |  |  |  |  |  |  |
| 06 |  |  |  |  |  |  |  |  |  |  |
| 07 |  |  |  |  |  |  |  |  |  |  |
| 08 |  |  |  |  |  |  |  |  |  |  |
| 09 |  |  |  |  |  |  |  |  |  |  |
| 10 |  |  |  |  |  |  |  |  |  |  |

| **SECOND VISIT (review or referral)** | | | | | | | | | |
| --- | --- | --- | --- | --- | --- | --- | --- | --- | --- |
| ID | **E35**  Was there a well organized and fair queuing system at the health facility?  **Yes…….1**  **No………2** | **E36**  Were there sufficiently doctors/medical assistants at the health facility?  **Yes…………1**  **No…………2** | **E37**  Did the Doctors/Medical Assistants/Nurses treat you [NAME] respectfully?  **Yes…………….1**  **No………………2** | **E38**  Did you [NAME] get a prescription for drugs at this facility?  **Yes.............1**  **No..............2**  **if No skip to E44** | **E39**  Did you [NAME] receive all prescribed drugs from this facility?  **Yes, all drugs............1**  **Some drugs............2**  **None of the drugs………….3**  **If 3 skip to E43** | **E40**  Did you [name] pay for drugs received at the facility during this visit?  **Yes………. 1**  **No…………2**  **If no, skip to E42** | **E41**  How much did you [NAME] pay for drugs?  **AMOUNT IN GH CEDIS** | **E42**  Why did you [NAME] not pay for drugs you/he/she received?  **Used NHIS card..............1**  **Could not afford.............2**  **Covered by employer.....3**  **Other, specify…………...99** | **E43**  Did you [NAME] pay for drugs elsewhere?  **yes........1**  **No………2**  **AMOUNT IN GH CEDIS**  **If no, skip to E45** |
| 01 |  |  |  |  |  |  |  |  |  |
| 02 |  |  |  |  |  |  |  |  |  |
| 03 |  |  |  |  |  |  |  |  |  |
| 04 |  |  |  |  |  |  |  |  |  |
| 05 |  |  |  |  |  |  |  |  |  |
| 06 |  |  |  |  |  |  |  |  |  |
| 07 |  |  |  |  |  |  |  |  |  |
| 08 |  |  |  |  |  |  |  |  |  |
| 09 |  |  |  |  |  |  |  |  |  |
| 10 |  |  |  |  |  |  |  |  |  |

| **SECOND VISIT (review or referral)** | | | **Now I would like to ask you questions about Complaint Handling about the Health Facilities in your Community.** | | | | | |
| --- | --- | --- | --- | --- | --- | --- | --- | --- |
| **ID** | **E44**  How much did you [NAME] pay for drugs elsewhere?  **AMOUNT IN GH CEDIS** | **E45**  How much did you [NAME] pay for transportation in total for this first visit including transport for getting medicines?  **AMOUNT IN GH CEDIS** | **E46**  If you have a complaint about your health facility, where do you go?  **Management of facility………………..…1**  **NHIS Office/Agent..2**  **Chief’s Palace……….3**  **District Assembly....4**  **Community leader..5**  **Other specify………99**  **MULTIPLE RESPONSES ALLOWED** | **E47**  In the last 12 months, did you have any complaints about the health facilities in your community?  **Yes……………...1**  **No……………….2**  **If No, end section** | **E48**  What was the complaint about?  **Long waiting time……..1**  **No drugs at facility……………………...2**  **Have NHIS card but paid money……………...3**  **Nurses rude……………..4**  **Unfair queuing system……………………..5**  **Other specify………....99** | **E49**  Did you report this complaint?  **Yes…………1**  **No………….2**  **If No end section** | **E50**  Where did you report this complaint?  **Management of facility………………1**  **NHIS Office……….2**  **Chief’s Palace……3**  **District Assembly............4**  **Community leader……………….5**  **Other specify……99** | **E51**  How was the complaint handled?  Very well....1  Well……….…2  Good ……….3  Bad…………..4  Very Bad…..5 |
| 01 |  |  |  |  |  |  |  |  |
| 02 |  |  |  |  |  |  |  |  |
| 03 |  |  |  |  |  |  |  |  |
| 04 |  |  |  |  |  |  |  |  |
| 05 |  |  |  |  |  |  |  |  |
| 06 |  |  |  |  |  |  |  |  |
| 07 |  |  |  |  |  |  |  |  |
| 08 |  |  |  |  |  |  |  |  |
| 09 |  |  |  |  |  |  |  |  |
| 10 |  |  |  |  |  |  |  |  |

| **CODES FOR QUESTION E9b** | | | | | |
| --- | --- | --- | --- | --- | --- |
| MALARIA / FEVER………......................... 1 | | WORMS/PARASIT……..8 | SKIN DISEASES…………….15 | | HYPERTENSION....22 |
| PREGNANCY COMPLICATION……………...2 | | EAR, NOSE, THROAT.....9 | HEART DISEASE……….….16 | | BURNS………………23 |
| EYE INFECTION......................................3 | | SNAKE/DOG BITE……..10 | INJURY……………………….17 | | POISONING..........24 |
| RESPIRATORY TRACT INFECTION…….…..4 | | FRACTURES……………….11 | DENTAL PROBLEM……..18 | | ANEMIA...............25 |
| ANTE-NATAL CARE……………………………...5 | | TB...............................12 | DELIVERY OF CHILD…….19 | | DIARRHEA............26 |
| REPRODUCTIVE TRACT INFECTION………6 | | CONVULSION……………13 | Cough…….………………….20 | | OTHER…………………27 |
| POST-NATAL CARE……………………………...7 | | PREVENTIVE HEALTH..14 | IMMUNIZATION…………21 | | FAMILY PLANNING…28 |
| **Codes FOR Question E10** | | | | | |
| TEACHING HOSPITAL……………………1 | PRIVATE CLINIC …………………………..8 | | | TRADITIONAL HEALER HERBALIST……..15 | |
| REGIONAL PUBLIC HOSPITAL ……….2 | PRIVATE HOSPITAL………………………..9 | | | SPIRITUALIST……………………………………..16 | |
| DISTRICT PUBLIC HOSPITAL ………….3 | PHARMACY…………………………….…..10 | | | DRUG PEDLERS…………………………………..17 | |
| MISSION CLINIC……………………...4 | PRIVATE DOCTOR/NURSE………..…11 | | | CHEMICAL SELLER……………………………...18 | |
| CLINIC AT WORK…………………………..5 | PRIVATE MIDWIFE……………………...12 | | | TRADITIONAL BIRTH ATTENDANT……..19 | |
| CHPS COMPOUND……………………….6 | PRIVATE DENTIST………………………..13 | | | OTHER SPECIFY………………………………….20 | |
| HEALTH CENTER…………………………..7 | COMMUNITY HEALTH WORKER….14 | | |  | |

|  | **SECTION F: NHIS ENROLMENT**  I would like to ask you about your enrollment and perceptions of the NHIS  RESPONDENT: HOUSEHOLD HEAD FOR ALL HOUSEHOLD MEMBERS | | | | | | | | | | |
| --- | --- | --- | --- | --- | --- | --- | --- | --- | --- | --- | --- |
| **ID** | **F 1a**  Are your/ [NAME] neighbors currently enrolled in any health insurance scheme?  **Yes......1**  **No.......2** | **F1b**  Are you / [NAME] currently enrolled in any health insurance scheme?  **Yes......1**  **No.......2**  **If Yes, skip to F2c** | **F2a**  Have you / Has [NAME] ever been enrolled in the NHIS?  **Yes, Previously enrolled……1**  **No, Never enrolled…..2**  **If Yes, skip to F3** | **F2b**  What is the one main reason why you [NAME] have never been insured in the NHIS?  Cannot afford premium.............1  Never heard of NHIS..................2  Covered by employer.................3  Mostly healthy do not need NHIS………………………………………..4  No scheme in the area...............5  No confidence in the scheme….6  Registration point too far..........7  Have private health insurance…8  Others specify...........................99  **If answered end section** | **F2c**  What type of scheme are you / [NAME] currently enrolled in?  **NHIS……..1**  **Private…..2**  **Other specify…..3**  **Skip to F4 if answered** | **F3**  When did your/ [NAME] registration end?  **Month/ YEAR**  **Eg.03/08**  **End section if answered** | **F4**  Since when have you / has [NAME] been enrolled?  **MONTH/ YEAR**  **Eg 03/08** | **F 5**  Are you / [NAME] exempted from paying premium?  **Yes………1**  **No……….2**  **If Yes, skip to F8** | **F6**  How much premium do you / [NAME] pay per year?  **AMOUNT IN GH CEDI** | **F7**  How much registration fees did you / [NAME] pay on your first registration?  **AMOUNT IN GH CEDI** | **F8**  Why are you  exempted from  paying  premium?  **Aged 70+…..……1**  **Under 18 yrs……2**  **Pregnant**  **Woman…………..3**  **SSNIT contrib….4**  **Indigent ………….5** |
| **01** |  |  |  |  |  |  |  |  |  |  |  |
| **02** |  |  |  |  |  |  |  |  |  |  |  |
| **03** |  |  |  |  |  |  |  |  |  |  |  |
| **04** |  |  |  |  |  |  |  |  |  |  |  |
| **05** |  |  |  |  |  |  |  |  |  |  |  |
| **06** |  |  |  |  |  |  |  |  |  |  |  |
| **07** |  |  |  |  |  |  |  |  |  |  |  |
| **08** |  |  |  |  |  |  |  |  |  |  |  |
| **09** |  |  |  |  |  |  |  |  |  |  |  |
| **10** |  |  |  |  |  |  |  |  |  |  |  |

| **ID** | **F9**  Did you [NAME] receive the NHIS card for this last registration?  **Yes................1**  **No.................2**  **If No, skip to F16** | **F10**  How long did you [NAME] have to wait to receive this card?  **RECORD NUMBER OF MONTHS** | **F11**  When was your [NAME] last renewal date?  **MONTH/ YEAR**  **Eg 03/08** | **F12**  What was the actual date that you [NAME] last renewed your NHIS membership?  **MONTH/ YEAR**  **Eg 03/08** | **F13**  If you did not renew on the renewal date what was the reason for the delay?  **No Money for premium………...1**  **NHIS office too far…………………2**  **Agent did not come…………......3**  **Did not know where to go……..4**  **Not satisfied with provider…….5**  **Did not use service last yr………6**  **Health facility too far…………….7**  **Other specify………………………99** | **F14**  Where did you last renew your [NAME] NHIS membership?  **NHIS district office………1**  **Nearest health facility…2**  **Agent in community……3**  **Other specify……………..99** | **F15**  How far is your [NAME] house from this NHIS renewal point?  **RECORD IN KILOMETERS** |
| --- | --- | --- | --- | --- | --- | --- | --- |
| **01** |  |  |  |  |  |  |  |
| **02** |  |  |  |  |  |  |  |
| **03** |  |  |  |  |  |  |  |
| **04** |  |  |  |  |  |  |  |
| **05** |  |  |  |  |  |  |  |
| **06** |  |  |  |  |  |  |  |
| **07** |  |  |  |  |  |  |  |
| **08** |  |  |  |  |  |  |  |
| **09** |  |  |  |  |  |  |  |
| **10** |  |  |  |  |  |  |  |

| **FOR HOUSEHOLDS MEMBERS THAT ARE ABOVE 18 YEARS AND CURRENTLY INSURED WITH THE NHIS** | | | | | | | | | |
| --- | --- | --- | --- | --- | --- | --- | --- | --- | --- |
| **ID** | **F16**  From which main source did you hear about the NHIS?  **Health care facility..1**  **Radio......................2**  **TV..........................3**  **Health Insurance agent.....................4**  **From a friend.........5**  **From a relative......6**  **At work..................7**  **Church…………………8**  **Other specify.......99** | **F17**  What is the most convenient way of premium payment for you?  **Once a year……….1**  **Twice a year………2**  **Three times a yr…3**  **Four times a yr…..4**  **Other specify……………99** | **F17**  What is the most convenient time for premium payment for you?  **Beginning of yr…1**  **Second quarter…2**  **Third quarter……3**  **End of the yr…….4**  **Other specify……99** | **F18**  If you have a complaint about the NHIS, where do you go?  **NHIS District Office…………………..1**  **Chief’s Palace………2**  **District Assembly...3**  **Community leader.4**  **Other specify……..99** | **F19**  In the last 12 months, did you have any complaints about the NHIS office in your community?  **Yes……..1**  **No………2**  **If No, end section** | **F20**  What was the complaint about?  **Did not receive NHIS card………..1**  **Lost NHIS card………………..2**  **Have NHIS card but paid for service……………3**  **Have NHIS card but paid for drugs………………4**  **Other specify….99** | **F21**  Did you report this complaint?  **Yes….1**  **No……2**  **If No end section** | **F22**  Where did you report this complaint?  **NHIS District Office……………1**  **Chief’s Palace…………..2**  **District Assembly………3**  **Community leader…………..4**  **Other specify.99** | **F23**  How was the complaint handled?  **Very well...... 1**  **Well…… 2**  **Good …. 3**  **Bad……. 4**  **Very Bad 5** |
| **01** |  |  |  |  |  |  |  |  |  |
| **02** |  |  |  |  |  |  |  |  |  |
| **03** |  |  |  |  |  |  |  |  |  |
| **04** |  |  |  |  |  |  |  |  |  |
| **05** |  |  |  |  |  |  |  |  |  |
| **06** |  |  |  |  |  |  |  |  |  |
| **07** |  |  |  |  |  |  |  |  |  |
| **08** |  |  |  |  |  |  |  |  |  |
| **09** |  |  |  |  |  |  |  |  |  |
| **10** |  |  |  |  |  |  |  |  |  |

| **ID** | **F24**  Do you currently have enough information on the NHIS benefit package?  **Yes……….1**  **No………..2**  **If No, skip to F26** | **F25**  From which one main source did you obtain this benefit package information?  **Health care facility..1**  **Radio......................2**  **TV...........................3**  **Health Insurance agent......................4**  **From a friend..........5**  **From a relative.......6**  **At work...................7**  **Church………………….8**  **Other specify........99** | **F26**  Do you currently have sufficient information about NHIS premium level and exemption policy?  **Yes…….1**  **No……..2**  **If No, skip to F28** | **F27**  From which one main source did you obtain this premium level and exemption policy information?  **Health care facility…………1**  **Radio...............................2**  **TV....................................3**  **Health Insurance agent....4**  **From a friend...................5**  **From a relative................6**  **At work............................7**  **Church…………………………..8**  **Other specify..................99** | **F28**  Do you currently have sufficient information about NHIS registration and renewal processes?  **Yes…….1**  **No……..2**  **If No, skip to F30** | **F29**  From which one main source did you obtain this registration and renewal information?  **Health care facility..1**  **Radio.....................2**  **TV..........................3**  **Health Insurance agent....................4**  **From a friend........5**  **From a relative......6**  **At work.................7**  **Church………………..8**  **Other specify.......99** | **F30**  Do you currently have sufficient information about medicines that are covered under the NHIS?  **Yes…….1**  **No……..2**  **If No, skip to F32** | **F31**  From which one main source did you obtain this NHIS medicine coverage information?  **Health care facility..1**  **Radio......................2**  **TV...........................3**  **Health Insurance agent......................4**  **From a friend..........5**  **From a relative.......6**  **At work...................7**  **Church………………….8**  **Other specify........99** |
| --- | --- | --- | --- | --- | --- | --- | --- | --- |
| **01** |  |  |  |  |  |  |  |  |
| **02** |  |  |  |  |  |  |  |  |
| **033** |  |  |  |  |  |  |  |  |
| **04** |  |  |  |  |  |  |  |  |
| **05** |  |  |  |  |  |  |  |  |
| **06** |  |  |  |  |  |  |  |  |
| **07** |  |  |  |  |  |  |  |  |
| **08** |  |  |  |  |  |  |  |  |
| **09** |  |  |  |  |  |  |  |  |
| **10** |  |  |  |  |  |  |  |  |

| | **SECTION G: PERCEIVED QUALITY OF HEALTH CARE AT THE NHIS FACILITIES**  **Respondent: household head** | | | | | --- | --- | --- | --- | | **G1**  **a) What is the name of your nearest health facility?**  ……………………………………………………………. **INTERVIEWER: WRITE DOWN THE NAME OF THE FACILITY**  **b) How far away (km) is this facility from your house?**  **………………………………….km INTERVIEWER: WRITE DOWN DISTANCE IN KILOMETERS**  **c) How long does it take you to get to this facility (in minutes) from your house?**  **…………………………………... INTERVIEWER: WRITE DOWN TIME IN MINUTES** | | | | | **G2**  **What is your opinion on the following aspects of your nearest NHIS health facility** | | | | | **ID CODE** | a) The Docs./Med. Assistants/nurses are compassionate and very supportive | strongly agree 1|2 |3 |4 |5 strongly disagree |  | | b) The Docs./Med. Assistants/nurses treated me respectfully | strongly agree 1|2 |3 |4 |5 strongly disagree |  | | c) There are sufficiently good Docs./Med. Assistants/Nurses | strongly agree 1|2 |3 |4 |5 strongly disagree |  | | d) I don’t have to wait for a long time to see a doctor/medical assistant | strongly agree 1|2 |3 |4 |5 strongly disagree |  | | e) There is a well organized and fair queuing system | strongly agree 1|2 |3 |4 |5 strongly disagree |  | | d) I received all prescribed drugs from the facility | strongly agree 1|2 |3 |4 |5 strongly disagree |  | | e) There are adequate consulting rooms and medical equipment | strongly agree 1|2 |3 |4 |5 strongly disagree |  | | f) Health personnel treats patients with insurance cards in an equal way as patients without cards | strongly agree 1 |2 |3 |4 |5 strongly disagree |  | |  | g) I know where to go when I have a complaint about this facility | strongly agree 1 |2 |3 |4 |5 strongly disagree |  | |
| --- | --- | --- | --- | --- | --- | --- | --- | --- | --- | --- | --- | --- | --- | --- | --- | --- | --- | --- | --- | --- | --- | --- | --- | --- | --- | --- | --- | --- | --- | --- | --- | --- | --- | --- | --- | --- | --- | --- | --- | --- | --- |
|  |

| **G3**  **How satisfied are you about the following aspects of the nearest NHIS health facility** | | |
| --- | --- | --- |
| **ID CODE** | a) How satisfied are you with the services provided by the health facility? | very satisfied 1|2 |3 |4 |5 very dissatisfied |
|  | b) How satisfied are you with the information provided by the health facility? | very satisfied 1\|2 \|3 \|4 \|5 very dissatisfied |
|  | c) How satisfied are you with the place/desk for lodging complaints at the facility? | very satisfied 1\|2 \|3 \|4 \|5 very dissatisfied |
|  | d) How satisfied are you with the process of lodging complaint at the facility? | very satisfied 1\|2 \|3 \|4 \|5 very dissatisfied |
|  | e) How satisfied are you with the complaint handling and feedback by the health facility? | very satisfied 1\|2 \|3 \|4 \|5 very dissatisfied |
|  | f) How satisfied are you with the waiting time at the facility? | very satisfied 1\|2 \|3 \|4 \|5 very dissatisfied |

**SECTION H: PERCIEVED QUALITY OF NHIS SERVICE**

| **I would now make some statements about the NHIS and please ask you to respond using the 5 point score card I am going to show you. Please indicate whether you strongly agree, agree or strongly disagree with each statement.**  **RESPONDENT: HOUSEHOLD HEAD** | | | |
| --- | --- | --- | --- |
| **H1** | **What is your opinion on the following:** | | |
| ID | a) The information from media on NHIS is adequate | | strongly agree 1 |2 |3 |4 |5 strongly disagree |
|  | b)The NHIS is trustworthy | | strongly agree 1 \|2 \|3 \|4 \|5 strongly disagree |
|  | b)The services covered in the NHIS package are adequate | | strongly agree 1 \|2 \|3 \|4 \|5 strongly disagree |
|  | f) The 3 month waiting period for receiving ID card before accessing service is adequate | | strongly agree 1 \|2 \|3 \|4 \|5 strongly disagree |
|  | g) It is good that Schemes do not register children without their parents | | strongly agree 1 \|2 \|3 \|4 \|5 strongly disagree |
|  | h) The district scheme office opening hours are convenient | | strongly agree 1 \|2 \|3 \|4 \|5 strongly disagree |
|  | i) The district scheme office location is convenient | | strongly agree 1 \|2 \|3 \|4 \|5 strongly disagree |
|  | j) The distribution of NHIS cards is convenient | | strongly agree 1 \|2 \|3 \|4 \|5 strongly disagree |
|  | k) I know when to renew my membership | | strongly agree 1 \|2 \|3 \|4 \|5 strongly disagree |
|  | l) The premium for the NHIS package is too high | | strongly agree 1 \|2 \|3 \|4 \|5 strongly disagree |
|  | m) The registration fee is too high | | strongly agree 1 \|2 \|3 \|4 \|5 strongly disagree |
|  | n) The once a year premium payment is adequate | | strongly agree 1 \|2 \|3 \|4 \|5 strongly disagree |
| **H2** | **Indicate your level of satisfaction of the following aspect of the NHIS** | | |
|  | a) How satisfied are you with the services provided by the district NHIS office? | very satisfied 1|2 |3 |4 |5 very dissatisfied | |
|  | b) How satisfied are you with the information provided by the district NHIS office? | very satisfied 1\|2 \|3 \|4 \|5 very dissatisfied | |
|  | c) How satisfied are you about the complaint handling by the NHIS? | very satisfied 1\|2 \|3 \|4 \|5 very dissatisfied | |
|  | d) How satisfied are you about the benefit you derive from the NHIS? | very satisfied 1\|2 \|3 \|4 \|5 very dissatisfied | |
|  | e) How satisfied are you about the medicine list under the NHIS? | very satisfied 1\|2 \|3 \|4 \|5 very dissatisfied | |
|  | f) How satisfied are you about the category of people on the exemption package under the NHIS? | very satisfied 1\|2 \|3 \|4 \|5 very dissatisfied | |
|  | g) How satisfied are you with the distance from the NHIS office to your house? | very satisfied 1\|2 \|3 \|4 \|5 very dissatisfied | |
|  | h) How satisfied are you with the NHIS registration and renewal process? | very satisfied 1\|2 \|3 \|4 \|5 very dissatisfied | |

| **H3** | **Indicate you level of agreement or disagreement with the following general statements and perceptions of the NHIS?** | |
| --- | --- | --- |
|  | a) The NHIS is good as my contribution can also help other people when they fall sick | strongly agree 1 |2 |3 |4 |5 strongly disagree |
|  | b) Health is a matter of fate (in hands of God) and NHIS cannot help me in dealing with its consequences | strongly agree 1 \|2 \|3 \|4 \|5 strongly disagree |
|  | c)Enrolling in the NHIS may bring bad luck and illness | strongly agree 1 \|2 \|3 \|4 \|5 strongly disagree |
|  | d)NHIS makes me independent as I do not need to ask for money elsewhere when my family falls ill | strongly agree 1 \|2 \|3 \|4 \|5 strongly disagree |
|  | e)Enrolling on the NHIS is a sign of being enlightened | strongly agree 1 \|2 \|3 \|4 \|5 strongly disagree |
|  | f)NHIS is something for the poor | strongly agree 1 \|2 \|3 \|4 \|5 strongly disagree |
|  | g)Spending money to enroll on the NHIS is a priority for me | strongly agree 1 \|2 \|3 \|4 \|5 strongly disagree |
|  | h)It is good to be member of the NHIS even if I don’t fall sick | strongly agree 1 \|2 \|3 \|4 \|5 strongly disagree |
|  | i) Only those who fall sick should register with the NHIS | strongly agree 1 \|2 \|3 \|4 \|5 strongly disagree |
|  | J)My children do not fall sick so I do not need the NHIS | strongly agree 1 \|2 \|3 \|4 \|5 strongly disagree |
|  | k)Opinion leaders in my community affect my decision to enroll in the NHIS | strongly agree 1 \|2 \|3 \|4 \|5 strongly disagree |
|  | l) My ability to pay the premium affects my decision to enroll in the NHIS | strongly agree 1 \|2 \|3 \|4 \|5 strongly disagree |
|  | m)Experience of others with the NHIS affects my decision to enroll | strongly agree 1 \|2 \|3 \|4 \|5 strongly disagree |
|  | n)The quality of health services I receive from the provider affects my decision to enroll in the NHIS | strongly agree 1 \|2 \|3 \|4 \|5 strongly disagree |
|  | o) The quality of the NHIS services at the district office affects my decision to enroll. | strongly agree 1 \|2 \|3 \|4 \|5 strongly disagree |
|  | p) Even though I did not fall sick this year, I will still have to renew my NHIS membership. | strongly agree 1 \|2 \|3 \|4 \|5 strongly disagree |

| **I TRUST AND SOLIDARITY**: I would like to ask you about trust and solidarity in your village/community  **RESPONDENT: HOUSEHOLD HEAD FOR ALL HOUSEHOLD MEMBERS** | | | | |
| --- | --- | --- | --- | --- |
| **1.1** | | **What is your opinion on the following statements about trust in your community/village? I trust……** | | |
|  |  | a) most people in this community. | | Strongly agree 1\|2 \|3 \|4 \|5 strongly disagree |
|  |  | b) my Traditional Chief | | Strongly agree 1\|2 \|3 \|4 \|5 strongly disagree |
|  |  | c) my village elders | | Strongly agree 1\|2 \|3 \|4 \|5 strongly disagree |
|  |  | d) my Assembly man/woman | | Strongly agree 1\|2 \|3 \|4 \|5 strongly disagree |
|  |  | e) the police | | Strongly agree 1\|2 \|3 \|4 \|5 strongly disagree |
|  |  | f) local politicians (DC) | | Strongly agree 1\|2 \|3 \|4 \|5 strongly disagree |
|  |  | g) national politicians | | Strongly agree 1\|2 \|3 \|4 \|5 strongly disagree |
| **I.2** | **What is your opinion on the following statements about solidarity in your community/village?** | | | |
|  | a) Most people in this community will help others when they are in need. | | Strongly agree 1\|2 \|3 \|4 \|5 strongly disagree | |
|  | b) One has to be alert in this community or will be taken advantage of. | | Strongly agree 1\|2 \|3 \|4 \|5 strongly disagree | |
|  | c) People in this community will contribute money to projects even if they don’t benefit directly. | | Strongly agree 1\|2 \|3 \|4 \|5 strongly disagree | |
|  | d) People in this community will spend time and energy on projects even if they don’t benefit directly. | | Strongly agree 1\|2 \|3 \|4 \|5 strongly disagree | |
|  | f) People in this community will collaborate to solve any health services related problem. | | Strongly agree 1\|2 \|3 \|4 \|5 strongly disagree | |

**SECTION I: SOCIAL CAPITAL**

| **II COLLECTIVE ACTION AND COOPERATION:**  I would like to ask you questions about **collective action and cooperation** | | | |
| --- | --- | --- | --- |
| **I.3** | **What is your response or opinion on the following questions and statements?** | | |
|  | a)Did you participate in any communal activity in your community in the last 12 months? | | Yes……1  No……..2  If **No,** skip to c) |
|  | b) How many times did you participate in communal activity in the last 12 months? | | No. of times……………. |
|  | c) Have your community members ever approached someone to help solve problems related to the quality or provision of Health Services? (e.g. poor attitude of staff) | | Yes……1  No…….2  If **No**, skip to e) |
|  | d) Who did they approach to help solve the problem? | | Traditional Leader………………….1  Health Provider…………………….2  NHIS Office………………………..3  Media (News Papers /Radio/TV)….4  No one…………………….……….5  Other specify………………………99 |
|  | f) Have your community members ever approached someone to help solve a problem related to quality of NHIS services? | | Yes……1  No…….2 |
|  | g) Who did they approach to help solve this problem | | Traditional Leader…………………1  Health Provider……………………2  NHIS Office……………………….3  Media (News Paper/Radio/TV)…..4  No one…………………………….5  Other specify………………………99 |
| **III INFORMATION AND COMMUNICATION:** I would like to ask you questions about information and communication in your community. | | | |
| **I.4** | | In which way do you get information about government activities in your community?  Name the 5 **most reliable** ways.  (If not clear, use an example like national immunization program) | Relatives, friends, neighbors……………….1  Traditional Chief ……………………….….2  Church Service/leaders………………..…...3  Newspapers…………………………….…..4  Groups/Associations……………………….5  Radio……………………………………….6  TV………………………………………….7  Community meetings………………………8  Internet………………………………..……9  Mobile phone………………………….…..10  Assembly men………………………….....11  Information service van…………………...12  Other Specify……………………………..99 |

| **IV. SOCIAL COHESION & INCLUSION: I will now ask you questions about social cohesion and inclusion in your community.** | | |
| --- | --- | --- |
| **I.5** | **I will now read out a number of statements about you and your community/village .Please tell me from each statement whether the statement applies to you to a very small extent or to a very great extent on a scale of 1 to 5.** | |
|  | a)I feel excluded because of my financial situation (example: excluded from social benefits of NHIS) | To a very great extent 1\|2\|3\|4\|5 to a very small extent |
|  | b) Because of my social status I get preferential treatment. | To a very great extent 1\|2\|3\|4\|5 to a very small extent |
|  | c) I feel excluded because of my ethnic background (example: excluded from social programs) | To a very great extent 1\|2\|3\|4\|5 to a very small extent |
|  | d) Because of my religious belief I receive more benefits than others (get preferential treatment). | To a very great extent 1\|2\|3\|4\|5 to a very small extent |
|  | e) Because of political alliance one gets preferential treatment. | To a very great extent 1\|2\|3\|4\|5 to a very small extent |
| **I.6** | Do you feel excluded or are you not taken seriously by the healthcare providers | Yes………………………1  No……………………….2  *If No, skip to I20* |
| **I7** | Due to what do you feel excluded? Due to your level of …  (multiple responses possible) | Education…………………………………….1  Landholding………………………………….2  Wealth………………… …………………….3  Social status………………………………….4  Religious beliefs…………………….……….5  Gender……………………………………….6  Generation…………………………………...7  political beliefs………………………….…...8  Ethnicity……………………………………..9  Languages…………………………………..10  Other specify………………………………..99 |
| **I8** | Have there been instances where these different preferential treatments led to disagreements in your community? | Yes……………………….1  No………………………..2  *If No, skip to I22* |
| **I9** | How are the problems caused by differences in treatment between community members handled in your community? | People work it out between themselves…………………………………1  Household members intervene…………………………………...2  Neighbors intervene………………………..3  Village leaders mediate…………………….4  Religious leaders mediate…………………..5  Judicial leaders mediate…………………….6  Other specify……………………………….99 |

| **V EMPOWERMENT AND POLITICS** | | |
| --- | --- | --- |
| **I.10** | **I will now read out a number of statements about empowerment and political action in your community/ Please tell me for each statement whether you strongly agree, agree, disagree or strongly disagree** | |
|  | a) I am very happy with my future prospects | strongly agree 1\|2 \|3 \|4 \|5 strongly disagree |
|  | b) I am able to make important decisions that can change the course of my life. | strongly agree 1\|2 \|3 \|4 \|5 strongly disagree |
| **I.11** | How many times were you able to jointly petition health care officials with community members for something beneficial to the community in the 12 months? | No. of times…. |
| **I.12** | Did you vote in the 2008 parliamentary and presidential elections? | Yes……………………….1  No………………………..2 |
| **I.13** | Are you intending to vote in the 2012 parliamentary and presidential elections? | Yes……………………….1  No………………………..2 |

|  | **SECTION J: SOCIAL SCHEMES AND HEALTH SEEKING BEHAVIOR**  **Respondent: household head** | |
| --- | --- | --- |
| **J.1** | What is the most common cause of illness in this community?  **INTERVIEWER NOTE: one answer only** | A physical “worldly” cause (dirty environment, mosquitoes)………………………………………. 1  A spiritual cause ( as witches, gosts, ancestors)…….2  Other specify……………………………………….99 |
| **J.2** | Who in this household decides on whether or not somebody has to be sent to school?  **ALLOW UP TO 3 RESPONSES** | Head of HH…….…………...1  Spouse……..………………….2  Son/Daughter ……………..3  Son/Daughter in law…...4  Parent ……………..............5  Parent in law………………..6  Bro/Sis………..……………….7  Grandparents………………8  Other ………….……………..99 |
| **J.3** | Who in this household pays for the food?  **ALLOW UP TO 3 RESPONSES** | Head of HH…….…………...1  Spouse……..………………….2  Son/Daughter ……………..3  Son/Daughter in law…...4  Parent ……………..............5  Parent in law………………..6  Bro/Sis………..……………….7  Grandparents………………8  Other ………….……………..99 |
| **J.4** | When someone is ill in the household, who pays the healthcare bills  **ALLOW UP TO 3 RESPONSES** | Head of HH…….…………...1  Spouse……..………………….2  Son/Daughter ……………..3  Son/Daughter in law…...4  Parent ……………..............5  Parent in law………………..6  Bro/Sis………..……………….7  Grandparents………………8  Other ………….……………..99 |
| **J.5** | When someone is ill in this household , who takes care of the her/him?  **ALLOW UP TO 3 RESPONSES** | Head of HH…….…………...1  Spouse……..………………….2  Son/Daughter ……………..3  Son/Daughter in law…...4  Parent ……………..............5  Parent in law………………..6  Bro/Sis………..……………….7  Grandparents………………8  Other ………….……………..99 |

| **J.6** | When someone is ill, who in this household decides on the choice of a health care provider?  **ALLOW UP TO 3 RESPONSES** | Head of HH…….…………...1  Spouse……..………………….2  Son/Daughter ……………..3  Son/Daughter in law…...4  Parent ……………..............5  Parent in law………………..6  Bro/Sis………..……………….7  Grandparents………………8  Other ………….……………..99 |
| --- | --- | --- |
| **J.7** | In your household, what or who more influences the choice to go to a certain healthcare provider?  **ALLOW UP TO 2 RESPONSES** | Knowing the health provider/staff well….…………..1  Proximity of the facility……………………………………….2  Positive experience with the facility……………………3  Advise by a community member……….……………….4  Advise by the chief/elderly…..……….……………………5  Guidance by the NHIS staff…..……………………………..6  It is the only available health facility……………………7  Other specify……………………………………………………….99 |
| **J.8** | In your household, what or who more influences the choice to go and get enrolled in the NHIS  **ALLOW UP TO 2 RESPONSES** | Knowing the health insurance staff well..…………..1  Hearing positive experience of neighbors .…………2  Advise by a community member……….………………3  Advise by the chief/elderly…..……….…………………. 4  Other specify……………………………………………………….99 |

| **SECTION K : CONSUMPTION AND EXPENDITURES**  **RESPONDENT: HOUSEWIFE OR MOST INFORMED HOUSEHOLD MEMBER**    ***INSTRUCTION: ASK I2 FOR ALL ITEMS S inue the interview and FIRST, AND THEN ASK i3 AND I4 ONLY FOR THE ITEMS THAT WERE CONSUMED.*** | | |
| --- | --- | --- |
|  | **WEEKLY FOOD CONSUMPTION** |  |
| **K1** | Has your household consumed [FOOD ITEM] in the past seven days? | |
| | **item**  instruction: ask for all items first, and THEN ask J1a. & J1b. only for the items that were acquired | K1a. Has your household consumed [FOOD ITEM] in the past seven days?  Yes ………………..1  No………………….2 | K1b. How much did your household spend in total on [ITEM], including the value received for free?  **Quantity**  **Amount** | | | --- | --- | --- | --- | | 1. Rice |  |  |  | | 2. Corn |  |  |  | | 3. sugar |  |  |  | | 4. gari |  |  |  | | 5. yam |  |  |  | | 6. brodzi /Plantain |  |  |  | | 7.mankani/cocoyam |  |  |  | | 8. cassava |  |  |  | | 9. fish |  |  |  | | 10. meat |  |  |  | | 11. poultry |  |  |  | | 12. cooking oil |  |  |  | | 13. eggs |  |  |  | | 14. milk |  |  |  | | 15. salt |  |  |  | | 16. fruits |  |  |  | | 17. shrimps |  |  |  | | 18. vegetables |  |  |  | | 19. beans/Groundnuts |  |  |  | | 20. Bread/pastries |  |  |  | | 21. Tea |  |  |  | | 22. milo |  |  |  | | 23. coffee |  |  |  | | 24. cocoa powder |  |  |  | | 25. Butter/margarine |  |  |  | | 26. Alcoholic Drinks |  |  |  | | 27. .Non-Alcoholic Drinks |  |  |  | | 28. Cigarettes |  |  |  | | | |

|  | **MONTHLY NON-FOOD CONSUMPTION**  **RESPONDENT: HOUSEHOLD HEAD OR MOST INFORMED HOUSEHOLD MEMBER** | | | |
| --- | --- | --- | --- | --- |
| **K2** | Has your household purchased [ITEM] in the past 30 days or received it for free?. | | | |
|  | **item**  instruction: ask for all items first, and THEN ask J2a. & J2b. only for the items that were acquired | K2a. Has your household purchased [ITEM] in the past 30 days or received it for free?  Yes ………1  No………..2 | K2b. How much did your household spend in total on [ITEM], including the value received for free?  **Quantity Amount** | |
|  | 1. TOILET ARTICLES (soap, deodorant, powder, Troll, detol eg) |  |  |  |
|  | 2. FUEL (vehicle) |  |  |  |
|  | 3. TRAVEL |  |  |  |
|  | 4. INTEREST ON LOAN |  |  |  |
|  | 5. ELECTRICITY BILL |  |  |  |
|  | 6. PHONE BILL |  |  |  |
|  | 7. WATER BILL |  |  |  |
|  | 8. HOUSE RENT |  |  |  |
|  | 9. VEHICLE REPAIRS |  |  |  |
|  | 10. WOOD |  |  |  |
|  | 11. CHARCOAL |  |  |  |
|  | 12. GAS(kerosene) |  |  |  |
|  | **13.** MAINTENANCE AND REPAIR OF DWELLING AND UTILITIES |  |  |  |
|  | **14.** HOUSEHOLD OPERATION (detergents, insecticides, candles, batteries, etc.) |  |  |  |
|  | **15.** SERVICES AT BARBER, HAIR DRESSER, BEAUTY SALOONS |  |  |  |
|  | **17.** RESTAURANTS/CHOP BAR |  |  |  |

|  | **MONTHLY NON-FOOD CONSUMPTION**  **RESPONDENT: HOUSEHOLD HEAD OR MOST INFORMED HOUSEHOLD MEMBER** | | | | |
| --- | --- | --- | --- | --- | --- |
| **K3** | Has your household purchased [ITEM] in the **past 30 days** or received it for free? | | | | |
|  | **item**  instruction: ask for all items first, and THEN ask J3a. & J2b. only for the items that were acquired | | **K3a**. Has your household purchased [ITEM] in the past 30 days or received it for free?  Yes…………..1  No……………2 | **K3b**. How much did your household spend in total on [ITEM], including the value received for free?  **Quantity Amount** | |
|  | 1. CLOTHES | |  |  |  |
|  | 2. SLIPPERS/SHOES | |  |  |  |
|  | 3. SHEETS/BLANKETS/MATS | |  |  |  |
|  | 4. SCHOOL FEES/SCHOOL ITEMS | |  |  |  |
|  | 5. AGRICULTURE (INSTRUMENTS, FERTILIZER, PESTICIDES, SEEDS, WAGES) | |  |  |  |
|  | 6. INVESTMENT IN BUSINESS | |  |  |  |
|  | 7. LAND PAYMENT | |  |  |  |
|  | 8. BUILDING EXPENSES | |  |  |  |
|  | 9. HOUSE HELP (SECURITY, COOK, CLEANER ETC) | |  |  |  |
|  | 10. SAVINGS | |  |  |  |
|  | 11. TAX & INSURANCE | |  |  |  |
|  | 12. HEALTH (entire family) | |  |  |  |
|  | 13. REMITTANCES TO OTHERS | |  |  |  |
|  | 14. CHURCH DONATIONS | |  |  |  |
|  | 15. ENTERTAINMENT ( social events, funerals, out doorings, donations etc) | |  |  |  |
|  | 16. FURNITURE | |  |  |  |
|  | 17. BRIDE PRICE | |  |  |  |
|  | 18.PURCHASE OF VEHICLES(car, motorbike, bicycle) | |  |  |  |
|  | 19. PURCHASE OF ELECTRONIC EQUIPMENT(radio, tv,dvd refrigerators etc) | |  |  |  |
|  | **Question** | **Response** | | **CODE** | |
| **K4** | How would you rate the wealth of your household in comparison to others in this community? | Much poorer than most…………………………..1  Somewhat poorer than most........2  Similar to others....................................3  Richer than most........ 4  Much richer than most........... 5 | | |  | | --- | | |

|  |  | **To be recorded by the interviewer** |  |
| --- | --- | --- | --- |
| **No.** | **QUESTIONS AND FILTERS** | **RESPONSES** | **CODE** |
| **K5.a** | **INTERVIEWER: NOTE THE TYPE OF DWELLING**  ***RECORD OBSERVATION*** | Type of dwelling:  Single family house ……………………..………………..……….. 1  Apartment/flat ……………………………….……….…………….. 2  Rooms (compound house) ……………………….……………..3  Other (specify) ……….…………………………………………….... 99 | |  | | --- | |
| **K5.b** | **INTERVIEWER: NOTE The main roof material of the dwelling**  ***RECORD OBSERVATION*** | Thatch/straw ……………………………………………………………1  Wood …………………………………………………………………….2  Corrugated iron sheets …………………………………………..3  Cement ……………………………………………………………..…….4  Asbestos…………………………………………………………………..5  Brick Tiles …………………………………………………………6  Other [specify]………………….. ………………………….......... 99 | |  | | --- | |
| **K5.c** | **INTERVIEWER: Note the main floor material of the rooms**  ***RECORD OBSERVATION*** | Earth/sand…………………………...................................…1  Rudimentary /Wood planks……………………..……...…….2  Linoleum ……………………………………………………….. ..……3  Cement Blook………………………………………………………….4  Cement Tiles…..……………………………………………………...5  Wall-to-wall carpet……………….……………..………………….6  Other [specify] ………………..................................…….99 | |  | | --- | |
| **K5.d** | **INTERVIEWER: Note the material of walls in the house**  ***RECORD OBSERVATION*** | Earth/mud………………………….................................….1  Wood…………………………………………………….…...…….....2  Cement …..…………………………………………….………………3  Straw…..…………………………………………………………………4  Burnt bricks………………..……..…………………….……………5  Other [specify] ………………................................…….99 | |  | | --- | |
| **K5.e** | How many rooms does your household have?  **INTERVIEWER: EXCLUDE BATHROOM AND KITCHEN** | | Number of rooms | | --- | | |  |  | | --- | --- | |
| **K5.f** | What is the ownership status of your dwelling? | Built on squatter land……………………….……......…………..1  Given by relative or other rent-free ….……….....….......2  Provided by government rent-Free…………..…..….…....3  Rented……………………………………………………….…………….4  Owned ………………………………………………….….……………. 5  Other [Specify]……………………………………………………....99 | |  | | --- | |
| **K5.g** | Do you have electricity in your household? | Yes………………………..1  No………………………….2 | |  | | --- | |
| **K5.h** | What is the main source of drinking water in your household? | Indoor Plumbing…………………....................…..………..1  Inside Standpipe………………...................….…...........2  Water Vendor………………....................……….….........3  Water Truck/Tanker Service…………..…..…….............4  Neighboring Household………………….……………………..5  Public Standpipe………………..................….…..…........6  Well With Pump………………................…….….............7  Well Without Pump…………….................………….......8  River/Lake/Spring/Pond……...............……..….………..9  Rain Water…………....................……………..……….......10  Other [Specify]……...................……………..…………….99 | |  |  | | --- | --- | |
| **K5.i** | How long does it take to go get water (one way)? | HOUR…….. MINUTES………….. | |  |  | | --- | --- | |
| **K5.j** | What is the main fuel used by your household for cooking? | None……………………………………………………………………….0  Wood………………………………....................…..…….........1  Charcoal……………………….....................….….…………….2  Gas………………………………......................…………..........3  Electricity……………….....................………...….…………...4  Kerosene…………………………….........................……......5  Other [Specify]…………………..................…….....……….6 | |  | | --- | |
| **K5.k** | What kinds of toilet facilities do most members of your household use? | Flash Toilet…………………....................…...………………..1  Pit Latrine……………………....................…...……….........2  Pan/Bucket…………………...................…………...………..3  K.V.I.P……………………………….....................……….........4  No Toilet…………………………....................………...........5  Other [Specify]………………………...............................99 | |  | | --- | |
| **K5.l** | How many livestock does your household currently own?  Indicate number  (if more than one member of household owns livestock – add up) | | 1 | Cattle / Horse |  | | --- | --- | --- | | 2 | Sheep |  | | 3 | Goats |  | | 4 | Pigs |  | | 5 | Poultry |  | | 6 | Grass Cutters |  | | 99 | Other (Specify) |  | |  |

| **No.** | **QUESTIONS AND FILTERS** | **RESPONSES** | **QUESTIONS AND FILTERS** | **RESPONSES** |
| --- | --- | --- | --- | --- |
| **K6** | How many of the following items do you or any of your household members own in working condition?” And then just record the numbers. WRITE “0” IF ZERO. | **Quantity** | How many of the following items do you or any of your household members own in working condition?” And then just record the numbers. WRITE “0” IF ZERO | Quantity |
| | 1 | bed |  | 20. Car |  | | --- | --- | --- | --- | --- | | 2 | Living Room Furniture |  | 21.Floor Carpet |  | | 3 | Sewing machine |  | 22.Microwave |  | | 4 | Stove (Elec/ gas) |  | 23.Box iron |  | | 5 | Refrigerator |  | 24.Phone/ Mobile |  | | 6 | freezer |  | 25.Telephone Land line |  | | 7 | Fan |  | 26.Generator |  | | 8 | Radio/cassette |  | 27.Jewelry |  | | 9 | Record player |  | 28.Boat / Canoe |  | | 10 | VCR/DVD/VCD |  | 29.Tractor |  | | 11 | Computer |  |  |  | | 12 | Camcorder |  |  |  | | 13 | Satellite dish |  |  |  | | 14 | Washing machine |  |  |  | | 15 | TV |  |  |  | | 16 | Camera |  |  |  | | 17 | Iron (elec) |  |  |  | | 18 | Bicycle |  |  |  | | 19 | Motor cycle |  |  |  | | | | | |

| **No.** | **QUESTIONS AND FILTERS** | **RESPONSES** |
| --- | --- | --- |
| **K7** | How much land does your household own? | RECORD IN ACRES, BUT IF RESPONDENT CANNOT, USE SQUARE METRES OR LOCAL MEASUREMENTS (Poles or Ropes):  ……………………………………………Acres (1 acre=4plots)  …………………………………………...SQ. METRES  …………………………………………….FT  ……………………………………….…..Plots  ……………………………………………Poles |
|  | | |

***EVALUATION FORM***

***Please make sure to complete this section after the interview. It provides vital information for improving the survey instrument.***

| **Date (dd/mm/yy)** | **Location:** | | |  |
| --- | --- | --- | --- | --- |
| **Interviewer name** |  | | | |
|  | | | | |
| ***End Time:*** |  | | | |
| ***Are there questions that are unclear for the respondent?***  [ ] YES  [ ] NO | IF YES, which questions? | | | |
|  | Question number: | Remark: | | |
|  | Question number: | Remark: | | |
|  | Question number: | Remark: | | |
|  | Question number: | Remark: | | |
| ***Are there any words the respondent did not understand?***  [ ] YES  [ ] NO | If YES, which words? | | | |
|  |  |  |  |  |
|  |  |  |  |  |
|  |  |  |  |  |
| **Any other remarks or issues:** | | | | |

| **W would like to contact same household on our follow up survey, would you provide us details of three household members that could be contacted on our follow up survey** | | |
| --- | --- | --- |
| **id code** | **name** | **contact** |
|  |  |  |
|  |  |  |
|  |  |  |
