# Supplementary material for: Perceptions of healthcare quality in Ghana: Does health insurance status matter?
Source: PLoS One. 2018 Jan 16;13(1):e0190911. doi: 10.1371/journal.pone.0190911 (PMC5770037; doi:10.1371/journal.pone.0190911)
Supplement: S2 Appendix — (DOCX) [file pone.0190911.s002.docx]

**S1 Appendix.** **Effect of Currently Insured Status on Perceived Quality of Healthcare**

|  |  | **Average Perception** | **Service Provision** | **Complaint Lodging** | **Information Provision** | **Waiting Time** | **Prescribed Drugs** | **Equal Treatment** | **Queuing System** |
| --- | --- | --- | --- | --- | --- | --- | --- | --- | --- |
| **Strongly Disagree or Very Dissatisfied** | Insured | **-0.264*** | **-0.467***** | **-0.294**** | **-0.485***** | **-0.451***** | **-0.150** | **-0.346***** | **-0.285**** |
|  |  | (0.154) | (0.122) | (0.117) | (0.124) | (0.128) | (0.107) | (0.121) | (0.112) |
|  | Age | -0.006 | 0.003 | -0.002 | -0.003 | -0.002 | -0.009** | -0.002 | -0.004 |
|  |  | (0.004) | (0.004) | (0.004) | (0.004) | (0.004) | (0.003) | (0.004) | (0.004) |
|  | Female | -0.075 | -0.241** | -0.018 | -0.099 | 0.161 | -0.043 | -0.036 | 0.081 |
|  |  | (0.119) | (0.103) | (0.132) | (0.114) | (0.122) | (0.107) | (0.105) | (0.120) |
|  | Married | -0.191* | -0.492*** | -0.115 | -0.348*** | -0.124 | -0.088 | -0.201* | -0.203* |
|  |  | (0.108) | (0.102) | (0.119) | (0.097) | (0.101) | (0.099) | (0.119) | (0.122) |
|  | Christian | 0.113 | 0.056 | 0.049 | 0.142 | -0.227 | -0.109 | 0.180 | -0.112 |
|  |  | (0.142) | (0.148) | (0.184) | (0.136) | (0.139) | (0.147) | (0.129) | (0.135) |
|  | Household size | -0.026 | 0.010 | -0.011 | -0.017 | 0.019 | 0.032 | -0.017 | 0.016 |
|  |  | (0.025) | (0.027) | (0.031) | (0.028) | (0.027) | (0.028) | (0.026) | (0.029) |
|  | Rural | 0.133 | -0.130 | -0.136 | -0.029 | -0.260 | -0.095 | 0.117 | -0.093 |
|  |  | (0.229) | (0.134) | (0.150) | (0.131) | (0.174) | (0.174) | (0.157) | (0.155) |
|  | Facility visits | 0.056** | 0.036 | 0.051** | 0.013 | 0.009 | -0.025 | -0.002 | -0.000 |
|  |  | (0.024) | (0.037) | (0.025) | (0.027) | (0.031) | (0.027) | (0.026) | (0.031) |
|  | Primary Edu. plus | 0.375*** | 0.318*** | 0.093 | 0.330*** | 0.337*** | 0.242** | 0.309*** | 0.360*** |
|  |  | (0.115) | (0.116) | (0.232) | (0.117) | (0.117) | (0.103) | (0.099) | (0.115) |
|  | Work | -0.059 | 0.172 | 0.016 | -0.027 | -0.033 | -0.146 | -0.106 | -0.019 |
|  |  | (0.146) | (0.139) | (0.175) | (0.137) | (0.138) | (0.128) | (0.128) | (0.146) |
|  | _cons | 1.950*** | 0.708*** | 2.569*** | 1.341*** | 1.346*** | 1.376*** | 1.047*** | 0.823*** |
|  |  | (0.262) | (0.262) | (0.408) | (0.275) | (0.269) | (0.292) | (0.274) | (0.258) |
| **Disagree or Dissatisfied** | Insured | **-0.705***** | **-0.731***** | **-0.294**** | **-0.755***** | **-0.531***** | **-0.451***** | **-0.445***** | **-0.518***** |
|  |  | (0.131) | (0.145) | (0.117) | (0.140) | (0.112) | (0.108) | (0.105) | (0.124) |
|  | Age | -0.006 | 0.003 | -0.002 | -0.003 | -0.002 | -0.009** | -0.002 | -0.004 |
|  |  | (0.004) | (0.004) | (0.004) | (0.004) | (0.004) | (0.003) | (0.004) | (0.004) |
|  | Female | -0.075 | -0.241** | -0.018 | -0.099 | 0.161 | -0.043 | -0.036 | -0.275* |
|  |  | (0.119) | (0.103) | (0.132) | (0.114) | (0.122) | (0.107) | (0.105) | (0.163) |
|  | Married | -0.191* | -0.492*** | -0.115 | -0.348*** | -0.124 | -0.088 | -0.201* | -0.203* |
|  |  | (0.108) | (0.102) | (0.119) | (0.097) | (0.101) | (0.099) | (0.119) | (0.122) |
|  | Christian | 0.113 | 0.056 | 0.049 | 0.142 | -0.227 | -0.109 | 0.180 | -0.112 |
|  |  | (0.142) | (0.148) | (0.184) | (0.136) | (0.139) | (0.147) | (0.129) | (0.135) |
|  | Household Size | -0.026 | -0.102*** | -0.011 | -0.090*** | -0.045* | -0.009 | -0.051* | -0.091*** |
|  |  | (0.025) | (0.036) | (0.031) | (0.031) | (0.026) | (0.030) | (0.027) | (0.030) |
|  | Rural | -0.267 | -0.130 | -0.136 | -0.029 | -0.260 | -0.387** | -0.099 | -0.093 |
|  |  | (0.167) | (0.134) | (0.150) | (0.131) | (0.174) | (0.165) | (0.142) | (0.155) |
|  | No. Facility Visit | 0.056** | -0.005 | 0.051** | 0.013 | -0.042* | 0.028 | -0.002 | 0.041 |
|  |  | (0.024) | (0.034) | (0.025) | (0.027) | (0.024) | (0.027) | (0.026) | (0.027) |
|  | Primary Edu. Plus | 0.375*** | 0.318*** | -0.162 | 0.330*** | 0.337*** | 0.242** | 0.309*** | 0.360*** |
|  |  | (0.115) | (0.116) | (0.152) | (0.117) | (0.117) | (0.103) | (0.099) | (0.115) |
|  | Work | -0.059 | 0.172 | 0.016 | -0.027 | -0.033 | -0.146 | -0.106 | -0.019 |
|  |  | (0.146) | (0.139) | (0.175) | (0.137) | (0.138) | (0.128) | (0.128) | (0.146) |
|  | _cons | -0.103 | -0.800*** | 1.751*** | -0.408 | 0.132 | 0.395 | 0.116 | -0.529** |
|  |  | (0.272) | (0.252) | (0.396) | (0.258) | (0.262) | (0.277) | (0.272) | (0.269) |
| **Neutral** | Insured | **-0.088** | **-0.136** | **-0.294**** | **-0.248** | **-0.173** | **-0.100** | **-0.172** | **0.024** |
|  |  | (0.190) | (0.186) | (0.117) | (0.176) | (0.145) | (0.111) | (0.124) | (0.175) |
|  | Age | -0.006 | 0.003 | -0.002 | -0.003 | -0.002 | -0.009** | -0.002 | -0.004 |
|  |  | (0.004) | (0.004) | (0.004) | (0.004) | (0.004) | (0.003) | (0.004) | (0.004) |
|  | Female | -0.075 | -0.241** | -0.018 | -0.099 | 0.161 | -0.043 | -0.036 | -0.130 |
|  |  | (0.119) | (0.103) | (0.132) | (0.114) | (0.122) | (0.107) | (0.105) | (0.223) |
|  | Married | -0.191* | -0.492*** | -0.115 | -0.348*** | -0.124 | -0.088 | -0.201* | -0.203* |
|  |  | (0.108) | (0.102) | (0.119) | (0.097) | (0.101) | (0.099) | (0.119) | (0.122) |
|  | Christian | 0.113 | 0.056 | 0.049 | 0.142 | -0.227 | -0.109 | 0.180 | -0.112 |
|  |  | (0.142) | (0.148) | (0.184) | (0.136) | (0.139) | (0.147) | (0.129) | (0.135) |
|  | Household Size | -0.026 | -0.033 | -0.011 | 0.007 | 0.067** | 0.062** | 0.056** | -0.019 |
|  |  | (0.025) | (0.042) | (0.031) | (0.045) | (0.031) | (0.028) | (0.025) | (0.034) |
|  | Rural | -0.815*** | -0.130 | -0.136 | -0.029 | -0.260 | -0.278 | -0.047 | -0.093 |
|  |  | (0.308) | (0.134) | (0.150) | (0.131) | (0.174) | (0.199) | (0.177) | (0.155) |
|  | No. of Facility visits | 0.056** | 0.030 | 0.051** | 0.013 | 0.123*** | 0.102*** | -0.002 | 0.069*** |
|  |  | (0.024) | (0.030) | (0.025) | (0.027) | (0.029) | (0.025) | (0.026) | (0.026) |
|  | Primary Edu. Plus | 0.375*** | 0.318*** | 0.460* | 0.330*** | 0.337*** | 0.242** | 0.309*** | 0.360*** |
|  |  | (0.115) | (0.116) | (0.236) | (0.117) | (0.117) | (0.103) | (0.099) | (0.115) |
|  | Work | -0.059 | 0.172 | 0.016 | -0.027 | -0.033 | -0.146 | -0.106 | -0.019 |
|  |  | (0.146) | (0.139) | (0.175) | (0.137) | (0.138) | (0.128) | (0.128) | (0.146) |
|  | _cons | -2.495*** | -2.299*** | -2.488*** | -2.289*** | -1.683*** | -0.697** | -1.376*** | -1.960*** |
|  |  | (0.315) | (0.298) | (0.364) | (0.327) | (0.268) | (0.308) | (0.294) | (0.302) |
| **Agree or Satisfied** | Insured |  |  |  |  |  | **-0.069** | **-0.262** | **-0.118** |
|  |  |  |  |  |  |  | (0.166) | (0.161) | (0.282) |
|  | Age |  |  |  |  |  | -0.009** | -0.002 | -0.004 |
|  |  |  |  |  |  |  | (0.003) | (0.004) | (0.004) |
|  | Female |  |  |  |  |  | -0.043 | -0.036 | -0.349 |
|  |  |  |  |  |  |  | (0.107) | (0.105) | (0.285) |
|  | Married |  |  |  |  |  | -0.088 | -0.201* | -0.203* |
|  |  |  |  |  |  |  | (0.099) | (0.119) | (0.122) |
|  | Christian |  |  |  |  |  | -0.109 | 0.180 | -0.112 |
|  |  |  |  |  |  |  | (0.147) | (0.129) | (0.135) |
|  | Household Size |  |  |  |  |  | -0.011 | 0.026 | -0.087 |
|  |  |  |  |  |  |  | (0.051) | (0.037) | (0.070) |
|  | Rural |  |  |  |  |  | -0.584** | -0.466** | -0.093 |
|  |  |  |  |  |  |  | (0.241) | (0.233) | (0.155) |
|  | No. Facility Visit |  |  |  |  |  | 0.067** | -0.002 | 0.081*** |
|  |  |  |  |  |  |  | (0.032) | (0.026) | (0.027) |
|  | Primary Edu. plus |  |  |  |  |  | 0.242** | 0.309*** | 0.360*** |
|  |  |  |  |  |  |  | (0.103) | (0.099) | (0.115) |
|  | Work |  |  |  |  |  | -0.146 | -0.106 | -0.019 |
|  |  |  |  |  |  |  | (0.128) | (0.128) | (0.146) |
|  | _cons |  |  |  |  |  | -1.747*** | -2.147*** | -2.547*** |
|  |  |  |  |  |  |  | (0.309) | (0.320) | (0.329) |
|  | **No. of Obs.** | **1785** | **1768** | **1765** | **1774** | **1694** | **1778** | **1783** | **1783** |

Source: COHEiSION Project baseline survey (March 2012), N=1,903 household heads. Note: Standard errors in parenthesis are robust and corrected for clustering at the health facility level. *: p<0.10, **: p<0.05, ***: p<0.01.
