# Supplementary material for: Perceptions of healthcare quality in Ghana: Does health insurance status matter?
Source: PLoS One. 2018 Jan 16;13(1):e0190911. doi: 10.1371/journal.pone.0190911 (PMC5770037; doi:10.1371/journal.pone.0190911)
Supplement: S3 Appendix — (DOCX) [file pone.0190911.s003.docx]

**S2 Appendix.** **Association of Never Insured Status on Perceived Quality of Healthcare**

|  |  | **Average Perception** | **Service Provision** | **Complaint Lodging** | **Information Provision** | **Waiting Time** | **Prescribed Drugs** | **Equal Treatment** | **Queuing System** |
| --- | --- | --- | --- | --- | --- | --- | --- | --- | --- |
| **Strongly Disagree or Very Dissatisfied** | **Never insured** | **0.245** | **0.330**** | **0.026** | **0.294*** | **0.215** | **0.078** | **0.418**** | **0.038** |
|  |  | (0.153) | (0.164) | (0.174) | (0.157) | (0.150) | (0.153) | (0.171) | (0.143) |
|  | Age | -0.002 | 0.012** | -0.002 | -0.001 | -0.003 | -0.003 | 0.001 | -0.001 |
|  |  | (0.006) | (0.005) | (0.006) | (0.005) | (0.005) | (0.005) | (0.005) | (0.005) |
|  | Female | 0.085 | -0.105 | -0.030 | -0.036 | 0.257* | -0.115 | -0.009 | -0.033 |
|  |  | (0.143) | (0.134) | (0.156) | (0.131) | (0.138) | (0.150) | (0.120) | (0.164) |
|  | Married | -0.313*** | -0.591*** | -0.128 | -0.466*** | -0.151 | -0.247** | -0.158 | -0.391*** |
|  |  | (0.118) | (0.120) | (0.143) | (0.104) | (0.135) | (0.100) | (0.153) | (0.139) |
|  | Christian | -0.060 | -0.057 | -0.131 | 0.141 | -0.335* | -0.196 | 0.208 | -0.192 |
|  |  | (0.180) | (0.184) | (0.203) | (0.185) | (0.193) | (0.184) | (0.184) | (0.169) |
|  | Household Size | -0.050* | -0.019 | -0.038 | -0.015 | -0.040 | 0.012 | -0.034 | -0.020 |
|  |  | (0.027) | (0.037) | (0.040) | (0.035) | (0.035) | (0.032) | (0.032) | (0.035) |
|  | Rural | 0.199 | -0.041 | -0.013 | 0.050 | -0.162 | -0.065 | 0.088 | -0.131 |
|  |  | (0.268) | (0.142) | (0.170) | (0.145) | (0.179) | (0.186) | (0.153) | (0.176) |
|  | No. Facility Visit | 0.056* | -0.015 | 0.048 | -0.002 | 0.015 | 0.070** | 0.011 | 0.030 |
|  |  | (0.033) | (0.030) | (0.039) | (0.042) | (0.045) | (0.029) | (0.032) | (0.048) |
|  | Primary Edu. plus | 0.366** | 0.361*** | -0.079 | 0.239 | 0.405*** | 0.269** | 0.197 | 0.360*** |
|  |  | (0.143) | (0.137) | (0.178) | (0.150) | (0.153) | (0.128) | (0.132) | (0.136) |
|  | Work | -0.127 | 0.149 | -0.104 | -0.065 | -0.272 | 0.023 | 0.255 | -0.032 |
|  |  | (0.223) | (0.200) | (0.250) | (0.211) | (0.183) | (0.183) | (0.226) | (0.208) |
|  | _cons | 1.919*** | 0.312 | 3.032*** | 1.183*** | 1.664*** | 1.106** | 0.385 | 1.030*** |
|  |  | (0.448) | (0.387) | (0.546) | (0.428) | (0.417) | (0.463) | (0.407) | (0.386) |
| **Disagree or Dissatisfied** | **Never insured** | **0.245** | **0.709***** | **0.026** | **0.556***** | **0.215** | **0.129** | **0.451***** | **0.319*** |
|  |  | (0.153) | (0.174) | (0.174) | (0.142) | (0.150) | (0.156) | (0.161) | (0.182) |
|  | Age | -0.002 | 0.012** | -0.002 | -0.001 | -0.003 | -0.004 | 0.001 | -0.001 |
|  |  | (0.006) | (0.005) | (0.006) | (0.005) | (0.005) | (0.006) | (0.005) | (0.005) |
|  | Female | 0.085 | -0.105 | -0.030 | -0.036 | 0.257* | 0.094 | -0.009 | -0.033 |
|  |  | (0.143) | (0.134) | (0.156) | (0.131) | (0.138) | (0.142) | (0.120) | (0.164) |
|  | Married | -0.313*** | -0.591*** | -0.128 | -0.466*** | -0.151 | -0.247** | -0.158 | -0.391*** |
|  |  | (0.118) | (0.120) | (0.143) | (0.104) | (0.135) | (0.100) | (0.153) | (0.139) |
|  | Christian | -0.060 | -0.057 | -0.131 | 0.141 | -0.335* | -0.196 | 0.208 | -0.192 |
|  |  | (0.180) | (0.184) | (0.203) | (0.185) | (0.193) | (0.184) | (0.184) | (0.169) |
|  | Household Size | -0.050* | -0.144*** | -0.038 | -0.101*** | -0.078** | -0.038 | -0.064** | -0.126*** |
|  |  | (0.027) | (0.039) | (0.040) | (0.033) | (0.031) | (0.033) | (0.032) | (0.034) |
|  | Rural | -0.154 | -0.041 | -0.013 | 0.050 | -0.162 | -0.510*** | 0.088 | -0.131 |
|  |  | (0.186) | (0.142) | (0.170) | (0.145) | (0.179) | (0.174) | (0.153) | (0.176) |
|  | No. Facility Visit | 0.056* | -0.015 | 0.048 | -0.002 | -0.083** | 0.070** | 0.011 | 0.030 |
|  |  | (0.033) | (0.030) | (0.039) | (0.042) | (0.037) | (0.029) | (0.032) | (0.048) |
|  | Primary Edu. plus | 0.366** | 0.361*** | -0.079 | 0.239 | 0.405*** | 0.269** | 0.197 | 0.360*** |
|  |  | (0.143) | (0.137) | (0.178) | (0.150) | (0.153) | (0.128) | (0.132) | (0.136) |
|  | Work | -0.127 | 0.149 | -0.104 | -0.065 | -0.272 | 0.023 | -0.094 | -0.032 |
|  |  | (0.223) | (0.200) | (0.250) | (0.211) | (0.183) | (0.183) | (0.197) | (0.208) |
|  | _cons | -0.173 | -1.485*** | 2.040*** | -0.730* | 0.362 | 0.177 | -0.384 | -0.652 |
|  |  | (0.474) | (0.414) | (0.543) | (0.392) | (0.420) | (0.413) | (0.406) | (0.407) |
| **Neutral** | **Never insured** | **0.245** | **0.132** | **0.026** | **-0.159** | **0.215** | **-0.277*** | **0.060** | **-0.370** |
|  |  | (0.153) | (0.219) | (0.174) | (0.234) | (0.150) | (0.159) | (0.174) | (0.248) |
|  | Age | -0.002 | 0.012** | -0.002 | -0.001 | -0.003 | -0.017*** | 0.001 | -0.001 |
|  |  | (0.006) | (0.005) | (0.006) | (0.005) | (0.005) | (0.006) | (0.005) | (0.005) |
|  | Female | 0.085 | -0.105 | -0.030 | -0.036 | 0.257* | 0.106 | -0.009 | -0.033 |
|  |  | (0.143) | (0.134) | (0.156) | (0.131) | (0.138) | (0.161) | (0.120) | (0.164) |
|  | Married | -0.313*** | -0.591*** | -0.128 | -0.466*** | -0.151 | -0.247** | -0.158 | -0.391*** |
|  |  | (0.118) | (0.120) | (0.143) | (0.104) | (0.135) | (0.100) | (0.153) | (0.139) |
|  | Christian | -0.060 | -0.057 | -0.131 | 0.141 | -0.335* | -0.196 | 0.208 | -0.192 |
|  |  | (0.180) | (0.184) | (0.203) | (0.185) | (0.193) | (0.184) | (0.184) | (0.169) |
|  | Household Size | -0.050* | -0.034 | -0.038 | 0.002 | 0.057 | 0.097*** | 0.040 | -0.031 |
|  |  | (0.027) | (0.056) | (0.040) | (0.048) | (0.036) | (0.036) | (0.034) | (0.041) |
|  | Rural | -0.705** | -0.041 | -0.013 | 0.050 | -0.162 | -0.432* | 0.088 | -0.131 |
|  |  | (0.319) | (0.142) | (0.170) | (0.145) | (0.179) | (0.228) | (0.153) | (0.176) |
|  | No. Facility Visit | 0.056* | -0.015 | 0.048 | -0.002 | 0.148*** | 0.070** | 0.011 | 0.030 |
|  |  | (0.033) | (0.030) | (0.039) | (0.042) | (0.034) | (0.029) | (0.032) | (0.048) |
|  | Primary Edu. plus | 0.366** | 0.361*** | -0.079 | 0.239 | 0.405*** | 0.269** | 0.197 | 0.360*** |
|  |  | (0.143) | (0.137) | (0.178) | (0.150) | (0.153) | (0.128) | (0.132) | (0.136) |
|  | Work | -0.127 | 0.149 | -0.104 | -0.065 | -0.272 | 0.023 | 0.143 | -0.032 |
|  |  | (0.223) | (0.200) | (0.250) | (0.211) | (0.183) | (0.183) | (0.205) | (0.208) |
|  | _cons | -2.569*** | -2.688*** | -1.871*** | -2.134*** | -1.605*** | -0.295 | -1.793*** | -1.620*** |
|  |  | (0.499) | (0.476) | (0.514) | (0.498) | (0.433) | (0.414) | (0.436) | (0.444) |
| **Agree or Satisfied** | **Never insured** |  |  |  |  |  | **-0.633***** | **-0.002** | **-0.586** |
|  |  |  |  |  |  |  | (0.230) | (0.230) | (0.383) |
|  | Age |  |  |  |  |  | -0.030*** | 0.001 | -0.001 |
|  |  |  |  |  |  |  | (0.011) | (0.005) | (0.005) |
|  | Female |  |  |  |  |  | -0.366 | -0.009 | -0.033 |
|  |  |  |  |  |  |  | (0.279) | (0.120) | (0.164) |
|  | Married |  |  |  |  |  | -0.247** | -0.158 | -0.391*** |
|  |  |  |  |  |  |  | (0.100) | (0.153) | (0.139) |
|  | Christian |  |  |  |  |  | -0.196 | 0.208 | -0.192 |
|  |  |  |  |  |  |  | (0.184) | (0.184) | (0.169) |
|  | Household Size |  |  |  |  |  | 0.054 | 0.012 | -0.033 |
|  |  |  |  |  |  |  | (0.060) | (0.045) | (0.082) |
|  | Rural |  |  |  |  |  | -0.624** | 0.088 | -0.131 |
|  |  |  |  |  |  |  | (0.279) | (0.153) | (0.176) |
|  | No. Facility Visit |  |  |  |  |  | 0.070** | 0.011 | 0.030 |
|  |  |  |  |  |  |  | (0.029) | (0.032) | (0.048) |
|  | Primary Edu. plus |  |  |  |  |  | 0.269** | 0.197 | 0.360*** |
|  |  |  |  |  |  |  | (0.128) | (0.132) | (0.136) |
|  | Work |  |  |  |  |  | 0.023 | -0.329 | -0.032 |
|  |  |  |  |  |  |  | (0.183) | (0.286) | (0.208) |
|  | _cons |  |  |  |  |  | -0.618 | -2.308*** | -2.348*** |
|  |  |  |  |  |  |  | (0.594) | (0.520) | (0.575) |
|  | **No. of Obs.** | **1085** | **1074** | **1070** | **1080** | **1036** | **1079** | **1084** | **1083** |

Source: COHEiSION Project baseline survey (March 2012), N=1,903 household heads. Note: Standard errors in parenthesis are robust and corrected for clustering at the health facility level. *: p<0.10, **: p<0.05, ***: p<0.01
